# A Boost-Assisted Multi-Mode Bidirectional Resonant DC–DC Converter for Wide-Battery-Voltage-Range Applications

**DOI:** 10.3390/s26175606

**Published:** 2026-09-03

**Authors:** Wei Liu, Yilin Zhang, Li Cai, Tangbing Li, Fangming Deng, Yisheng Yuan, Zijian Zhou, Han Zeng

**Affiliations:** 1 School of Electrical and Automation Engineering, East China Jiaotong University, Nanchang 330013, China; 8502@ecjtu.edu.cn (W.L.); zy77656@gmail.com (Y.Z.); 2464@ecjtu.edu.cn (F.D.); 2465@ecjtu.edu.cn (Y.Y.); 3672@ecjtu.edu.cn (Z.Z.); 2 Nanchang Kechen Electric Power Test and Research Co., Ltd., Nanchang 330096, China; cailiclttt@163.com (L.C.); khanzerg@foxmail.com (T.L.)

**Keywords:** bidirectional DC–DC converter, LC resonance, soft switching, wide range

## Abstract

This paper proposes a bidirectional LC resonance DC–DC converter for wide-range applications. By introducing an auxiliary Boost bridge arm on the HVS of the conventional structure and multiplexing the resonant inductor, the proposed converter extends the voltage gain range. Utilizing a combined PWM and PFM control strategy, the converter operates in multiple modes: two gain modes (medium and high) during battery discharge, and three gain modes (low, medium, and high) during battery charging. This multi-mode mechanism effectively extends the voltage gain range, narrows the switching frequency variation, and simplifies magnetic component design. Furthermore, ZCS operation is confirmed under the tested representative operating conditions, significantly reducing switching losses. Finally, an experimental prototype with a rated power of 750 W was developed to verify the performance for 40–120 V battery charging and discharging requirements. The experimental results demonstrate the effectiveness and validity of the proposed topology and control strategy for high-efficiency, wide-range power conversion applications.

## 1. Introduction

Bidirectional DC–DC converters (BDCs) are critical for enabling bidirectional power flow and are widely used in microgrids, hybrid energy storage systems, and electric vehicles [1,2,3]. Typically, one port of a BDC is connected to a battery pack to accommodate different voltage levels, such as 40–60 V for residential energy storage systems, 50–120 V for electric tricycles, and 150–1000 V for electric passenger vehicles [4,5]. The other port is connected to the intermediate DC bus, with typical voltage levels of 200 V, 400 V, and 800 V. The wide range of battery operating voltages across these applications poses significant challenges for BDC design.

Common isolated bidirectional DC–DC (IBDC) converter topologies include dual-active-bridge (DAB) converters and resonant converters. DAB converters are widely utilized in medium- to high-power applications and are primarily based on phase-shift control to regulate voltage gain and power flow direction. Various control schemes, such as single-phase-shift (SPS) and multi-phase-shift (MPS) modulations, provide wide soft-switching and voltage gain ranges [6,7]. However, DAB converters exhibit high current stress and significant circulating power under wide voltage variations.

Typical resonant bidirectional DC–DC converter topologies include the LLC resonant converter [8,9] and the CLLC resonant converter [10]. The former provides a voltage gain greater than unity during forward operation; however, it effectively behaves as a series resonant converter in reverse mode, resulting in a gain of less than unity. Due to the use of pulse-frequency modulation (PFM), the wide switching frequency variation complicates the design of magnetic components. The latter resolves the issue of the LLC converter’s reverse gain being less than 1 but introduces an additional set of LC components on the low-voltage side for high-current applications, which significantly reduces the power density of the converter.

Conventional bidirectional LLC resonant converters utilizing PFM exhibit several limitations under wide input and output voltage conditions:

1. The wide switching frequency range inherent to PFM complicates the optimal design of magnetic components.
2. The limited voltage gain under heavy-load conditions makes it difficult to achieve a wide output voltage range.
3. At light loads or low voltage gains, the switching frequency becomes excessively high, leading to increased switching losses and reduced efficiency.

Consequently, conventional bidirectional LLC resonant converters are not well suited for applications requiring a wide input and output voltage range [11,12].

Existing methods to address the limited voltage gain under heavy-load conditions and reduced efficiency under light-load conditions in bidirectional LLC resonant DC–DC converters can be broadly classified into four categories.

The first category involves modifying resonant tank parameters [13,14], including auxiliary LC networks [13] or variable magnetizing inductance [14]. Auxiliary LC networks enable flexible tuning of resonant parameters and help reduce switching frequency variation but require additional switches. In contrast, variable magnetizing inductance allows the converter to operate in multiple modes to satisfy different gain requirements.

The second category focuses on varying the transformer turns ratio [15,16]. By employing reconfiguration switches to adjust the turns ratio, a wide output voltage range can be achieved. However, this approach increases copper losses in the transformer, reduces overall efficiency, and introduces significant voltage transients during switching, resulting in increased electrical stress.

The third category involves topology reconfiguration [17,18,19]. For example, a dual-resonant tank structure in [17] switches between single- and dual-tank modes to extend the gain range, but the mode transition introduces severe voltage spikes and requires complex control. In [18], an LCLL resonant tank with auxiliary inductors enables step-up capability during reverse operation; however, the reduced magnetizing inductance increases circulating power and degrades efficiency. In [19], a topology-morphing method switches between CLLC and CLLC-C configurations to achieve a wide gain range within a narrow frequency band, but the increased number of resonant components limits efficiency.

The fourth category employs advanced modulation strategies [20]. A hybrid modulation scheme combining pulse-frequency modulation (PFM) and phase-shift pulse-width modulation (PS-PWM) was proposed in [20], where PFM is used under heavy-load or high-voltage conditions, and PS-PWM is adopted under light-load or low-voltage conditions. Although this method constrains the switching frequency range and enables soft switching over a wide load range, it fails to address the low-efficiency issue under low-gain conditions and increases control complexity due to additional control variables.

Unlike existing reconfigurable resonant topologies that rely on additional resonant tanks or auxiliary inductors [17,18,19], the proposed five-arm structure achieves active boost capability by multiplexing the single resonant inductor *L*_r_. This structural integration liberates the transformer magnetizing inductance *L*_m_ from gain constraints, allowing *L*_m_ >> *L*_r_. As a result, circulating current is significantly suppressed compared with conventional LLC converters. Moreover, wide-range gain regulation is achieved predominantly at a fixed resonant frequency (*f*_s_ = *f*_r_) using PWM modulation in four of the five operating modes, effectively decoupling gain range extension from wide switching frequency variation. This converter is suitable for applications requiring bidirectional power exchange, such as battery energy storage systems.

## 2. Topology and Working Principle

**Figure 1:**
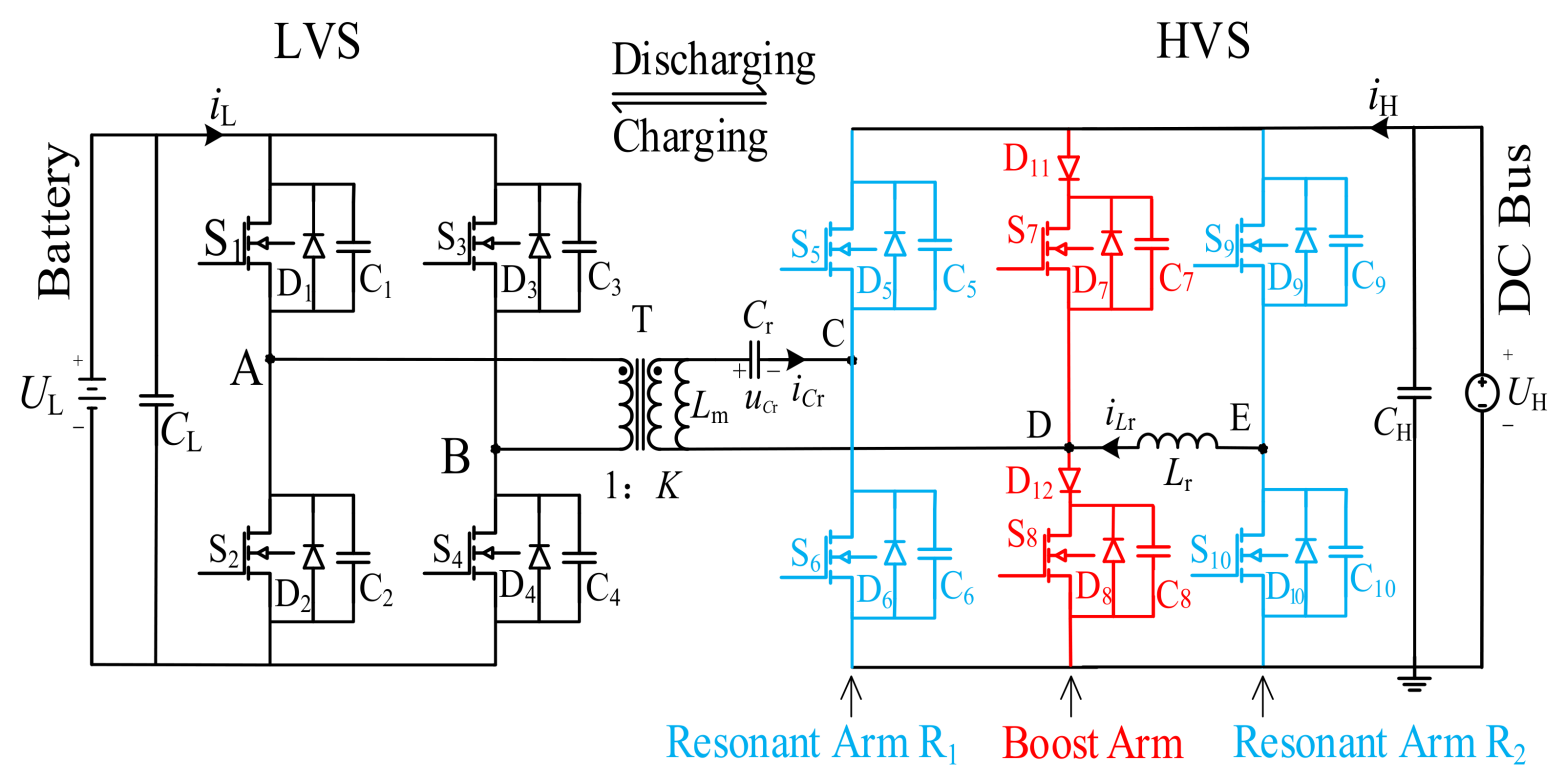
Proposed bidirectional DC–DC converter.

The proposed bidirectional DC–DC converter is illustrated in Figure 1. The low-voltage side (LVS), which serves as the battery port, adopts a full-bridge configuration composed of switches S_1_–S_4_. The high-voltage side (HVS), acting as the DC bus port, employs a three-arm structure. Specifically, switches S_5_–S_6_ and S_9_–S_10_ form the two resonant bridge arms, denoted as R_1_ and R_2_, respectively, while the boost arm is composed of the switch–diode pairs (S_7_, D_11_) and (S_8_, D_12_). Galvanic isolation between the two stages is achieved via a high-frequency transformer T with a turns ratio of 1:*K*.

By multiplexing the resonant inductor *L*_r_ and placing it on the HVS, where the current level is generally lower for a given transferred power, the proposed topology reduces the number of magnetic components and may facilitate a more compact magnetic design. As defined in Figure 1, *U*_L_ and *i*_L_ denote the LVS voltage and current, while *U*_H_ and *i*_H_ represent the HVS voltage and current. *C*_L_ and *C*_H_ are the filter capacitors on the LVS and HVS, respectively.

Compared with the conventional bidirectional LLC resonant DC–DC converter, the proposed topology introduces an additional boost arm on the HVS, allowing a larger transformer magnetizing inductance to be used and thereby reducing circulating current. This contributes to improved converter efficiency in both charging and discharging modes.

To simplify the analysis of the converter’s working principle, the following assumptions are considered:

1. *L*_m_ >> *L*_r_. Therefore, the magnetizing inductance *L*_m_ of transformer T is considered to be infinite, and the magnetizing current *i_L_*_m_ is approximately 0.
2. All switches, diodes, and capacitors are assumed to be ideal.

The following key parameters are defined: characteristic impedance $Z_{r}=\sqrt{L_{r}/C_{r}}$, quality factor $Q=\pi^{2}Z_{r}/\left(8K^{2}R_{L}\right)$, resonant frequency $f_{r}=1/(2\pi \sqrt{L_{r}C_{r}})$, switching frequency *f*_s_, and normalized frequency $f_{n}=f_{s}/f_{r}$ are defined, along with the resonant angular frequency $\omega_{r}=2\pi f_{r}$ and normalized angular frequency $\omega_{n}=\omega_{s}/\omega_{r}$.

The proposed converter operates in two voltage gain modes (high gain and medium gain) during battery discharge, while three distinct modes (high, medium, and low gain) are supported during the charging phase. These operational modes are elaborated in detail below.

### 2.1. Working Principle of Battery-Discharging Operation

During battery discharging, energy is transferred from the LVS to the HVS, with two operating modes, namely, the medium-gain mode and the high-gain mode. In addition, synchronous rectification is adopted on the HVS.

**Figure 2:**
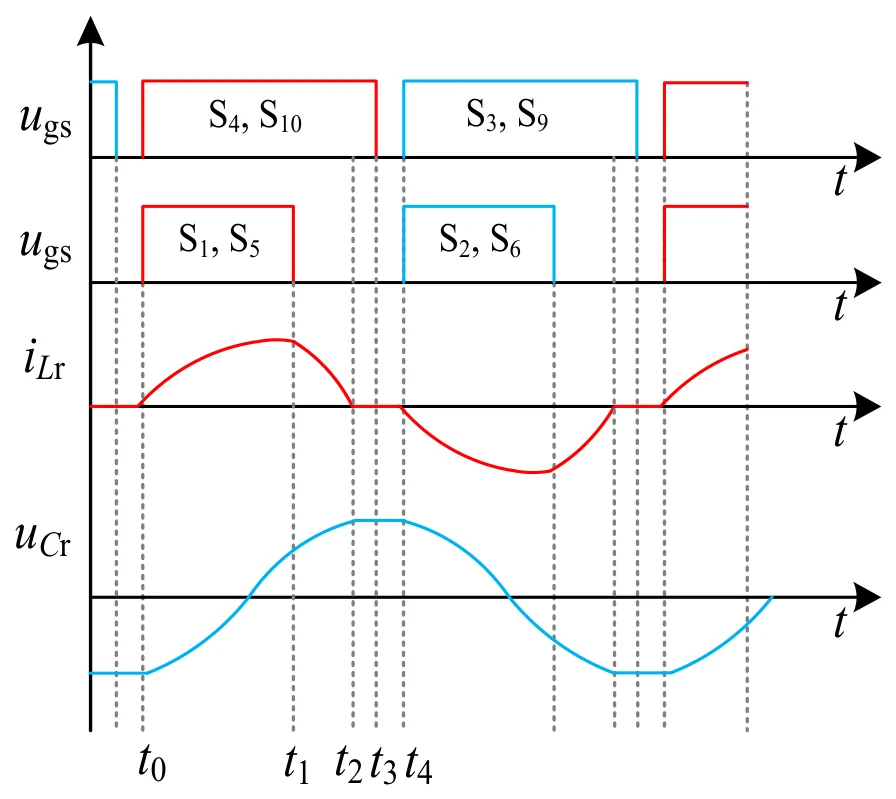
Main waveforms of DMG mode, where Stage 1, Stage 2, and Stage 3 correspond to the intervals [*t*_0_–*t*_1_), [*t*_1_–*t*_2_), and [*t*_2_–*t*_4_], respectively.

(1) Discharging Medium-Gain (DMG) Mode: When the battery voltage *U*_L_ is high, the converter operates in the DMG mode at *f*_s_ = *f*_r_. The corresponding key waveforms are shown in Figure 2, where *u*_gs_ represents the gate-drive voltages of the switches. On the LVS, switches S_1_ and S_2_ are PWM-controlled with a duty cycle of *D*_m_ = 2(*t*_1_ − *t*_0_)/*T*_s_, whereas S_3_ and S_4_ operate with a fixed duty cycle slightly lower than 0.5. Meanwhile, on the HVS, switches S_5_, S_6_, S_9_, and S_10_ are driven synchronously with the LVS switches S_1_, S_2_, S_3_, and S_4_, respectively.

**Figure 3:**
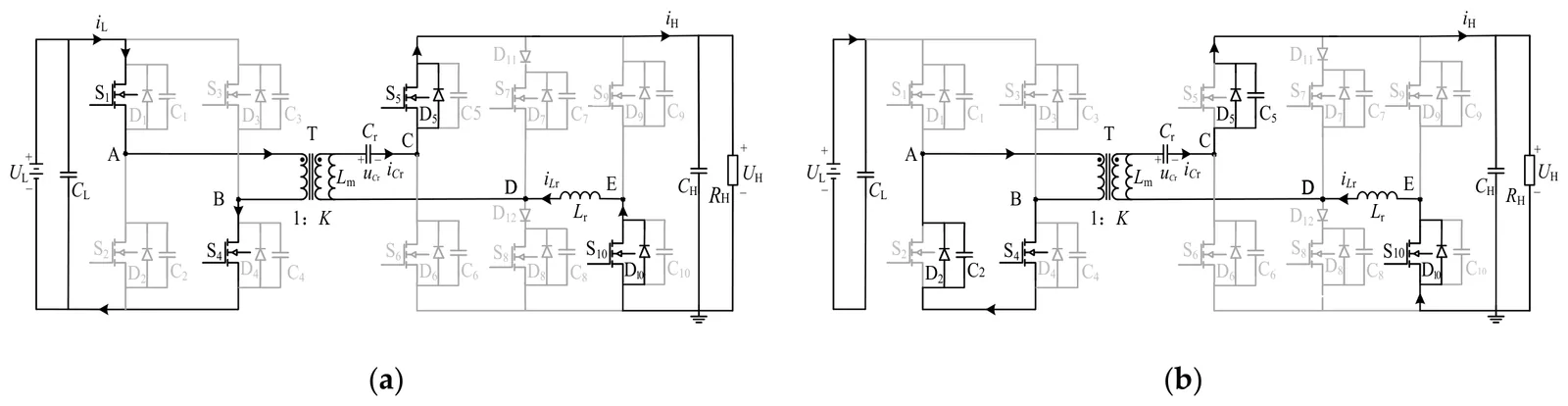
Equivalent circuits of each stage of DMG mode. (**a**) Stage 1. (**b**) Stage 2.

The interval [*t*_0_,*t*_4_] corresponds to three operating stages within the positive half-cycle, namely, the LC resonant stage, the circulating-current stage, and the current interruption and dead-time stage, as illustrated in Figure 3. A brief description of each stage is given as follows.

Stage 1 [*t*_0_–*t*_1_): LC resonant stage. At *t*_0_, the initial resonant inductor current *i_L_*_r_ is 0, and switches S_1_, S_4_, S_5_, and S_10_ are turned on under zero-current switching (ZCS). Meanwhile, the resonant capacitor voltage *u_C_*_r_ is at its negative peak, i.e., −Δ*U_C_*_rM_. Then, *L*_r_ and *C*_r_ start to resonate, and energy is transferred from the LVS to the HVS. During this stage, the time-domain expressions for *i_L_*_r_ and *u_C_*_r_ can be written as

(1) $$\left\{\begin{array}{l} i_{Lr}(t)=\frac{KU_{L}-U_{H}+\Delta U_{CrM}}{Z_{r}}\sin[\omega_{r}(t-t_{0})]\\ u_{Cr}(t)=KU_{L}-U_{H}-(KU_{L}-U_{H}+\Delta U_{CrM})\cos[\omega_{r}(t-t_{0})]\; \end{array}\right.$$

Stage 2 [*t*_1_–*t*_2_): Circulating-current stage. At *t*_1_, S_1_ is turned off, and D_2_ starts to conduct, causing the voltage *U_AB_* to be clamped to zero. The HVS circuit enters the circulating-current stage. During this interval, the voltage across *L*_r_ is −(*U*_H_ + *u_C_*_r_), which causes *i_L_*_r_ to decrease as the stored energy in the inductor is released.

Stage 3 [*t*_2_–*t*_4_]: Current interruption and dead-time stage. At *t*_2_, *i_L_*_r_ decreases to 0, indicating that the energy stored in *L*_r_ has been fully released, while *u_C_*_r_ reaches its positive peak value, Δ*U_C_*_rM_. At *t*_3_, switches S_4_ and S_10_ are turned off under ZCS. The interval [*t*_3_,*t*_4_] corresponds to the dead-time stage, during which the load is supplied by capacitor *C*_H_.

**Figure 4:**
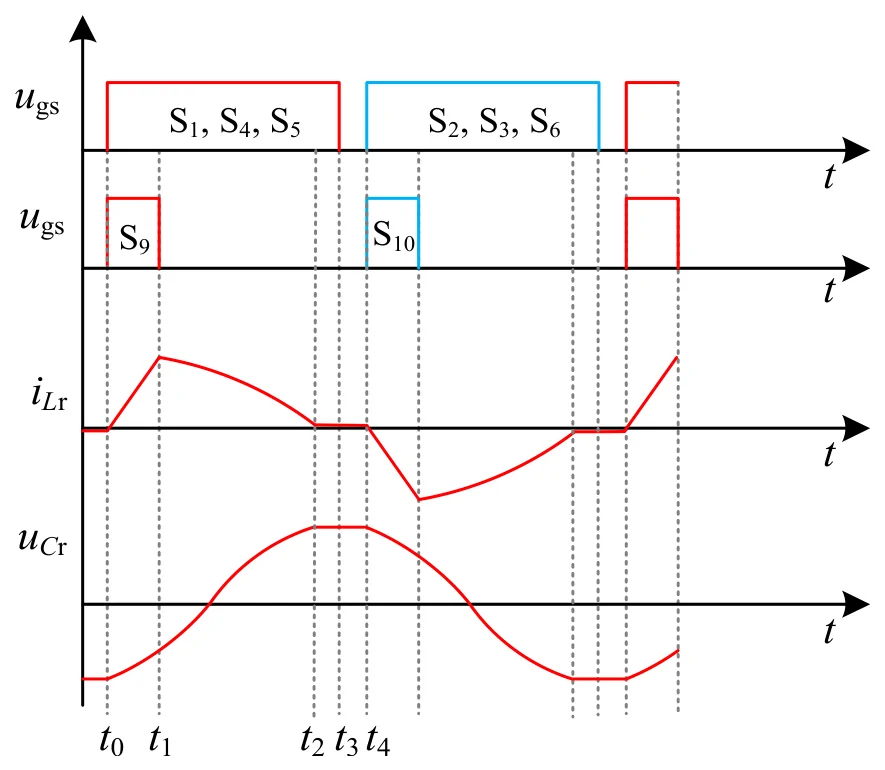
Main waveforms of DHG mode, where Stage 1, Stage 2, and Stage 3 correspond to the intervals [*t*_0_–*t*_1_), [*t*_1_–*t*_2_), and [*t*_2_–*t*_4_], respectively.

(2) Discharging High-Gain (DHG) Mode: When *U*_L_ is low, the converter operates in the DHG mode with *f*_s_ = *f*_r_. The corresponding key waveforms are shown in Figure 4. Switches S_9_ and S_10_ are controlled by PWM, with a duty cycle of *D*_h_ = 2(*t*_1_ − *t*_0_)/*T*_s_. The switch groups S_1_, S_4_, and S_5_ and S_2_, S_3_, and S_6_ operate with fixed duty cycles slightly lower than 0.5.

**Figure 5:**
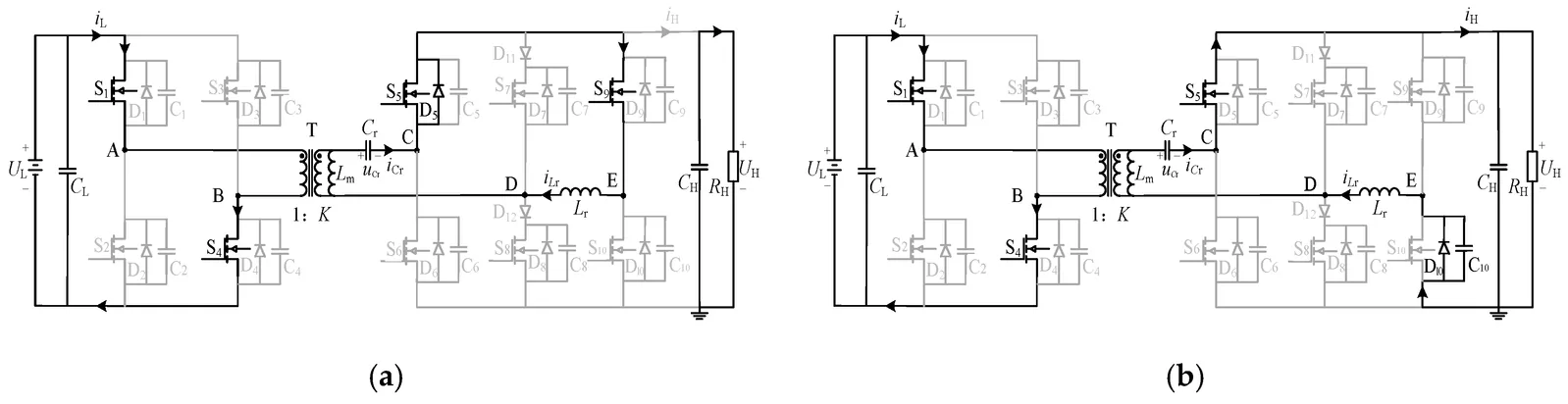
Equivalent circuits of each stage of DHG mode. (**a**) Stage 1. (**b**) Stage 2.

The interval [*t*_0_,*t*_4_] corresponds to three operating stages in the positive half-cycle, namely, the *L*_r_ energy storage stage, the LC resonant stage, and the current interruption and dead-time stage, as illustrated in Figure 5. A brief description of each stage is given as follows.

Stage 1 [*t*_0_–*t*_1_): *L*_r_ energy storage stage. At *t*_0_, the initial value of *i_L_*_r_ is 0, and switches S_1_, S_4_, S_5_, and S_9_ are turned on under ZCS. The initial voltage across *C*_r_ is −Δ*U_C_*_rM_, and the initial voltage across *L*_r_ is *KU*_i_ *+* Δ*U_C_*_rM_. Then, *L*_r_ and *C*_r_ resonate, and both the source *U*_L_ and capacitor *C*_r_ participate in energizing *L*_r_. During this stage, the time-domain expressions of *i_L_*_r_ and *u_C_*_r_ are given by

(2) $$\begin{array}{c} \left\{\begin{array}{l} i_{Lr}(t)=\frac{KU_{L}+\Delta U_{C\mathrm{rM}}}{Z_{r}}\sin[\omega_{r}(t-t_{0})]\;\\ u_{Cr}(t)=KU_{L}-(KU_{L}+\Delta U_{C\mathrm{rM}})\cos[\omega_{r}(t-t_{0})] \end{array}\right. \end{array}$$

During this stage, energy is stored in *L*_r_, while capacitor *C*_H_ supplies the load.

Stage 2 [*t*_1_–*t*_2_): LC resonant stage. At *t*_1_, switch S_9_ is turned off, marking the end of the *L*_r_ energy storage stage. The body diode D_10_ of switch S_10_ becomes forward-biased, and *L*_r_ and *C*_r_ start to resonate to transfer energy to the load. The initial value of the resonant current in this stage is *i_L_*_r_(*t*_1_). The time-domain expressions of *i_L_*_r_ and *u_C_*_r_ are given by

(3) $$\begin{array}{l} \left\{\begin{array}{l} i_{Lr}(t)=\frac{KU_{L}-U_{H}-u_{Cr}(t_{1})}{Z_{r}}\sin[\omega_{r}(t-t_{1})]+i_{Lr}(t_{1})\cos[\omega_{r}(t-t_{1})]\;\\ u_{Cr}(t)=KU_{L}-U_{H}+i_{Lr}(t_{1})Z_{r}\sin[\omega_{r}(t-t_{1})]+(KU_{L}-U_{H}-u_{Cr}(t_{1}))\cos[\omega_{r}(t-t_{1})] \end{array}\right. \end{array}$$

In this stage, energy is delivered to the load through LC resonance.

Stage 3 [*t*_2_–*t*_4_]: Current interruption and dead-time stage. At *t*_2_, *i_L_*_r_ decreases to zero. At *t*_3_, switches S_1_, S_4_, and S_5_ are turned off under ZCS. The interval [*t*_3_–*t*_4_] is the dead-time stage, during which capacitor *C*_H_ supplies the load.

### 2.2. Working Principle of Battery-Charging Operation

During battery charging, energy is transferred from the HVS to the LVS, with low-, medium-, and high-voltage gain modes. Synchronous rectification is adopted on the LVS.

**Figure 6:**
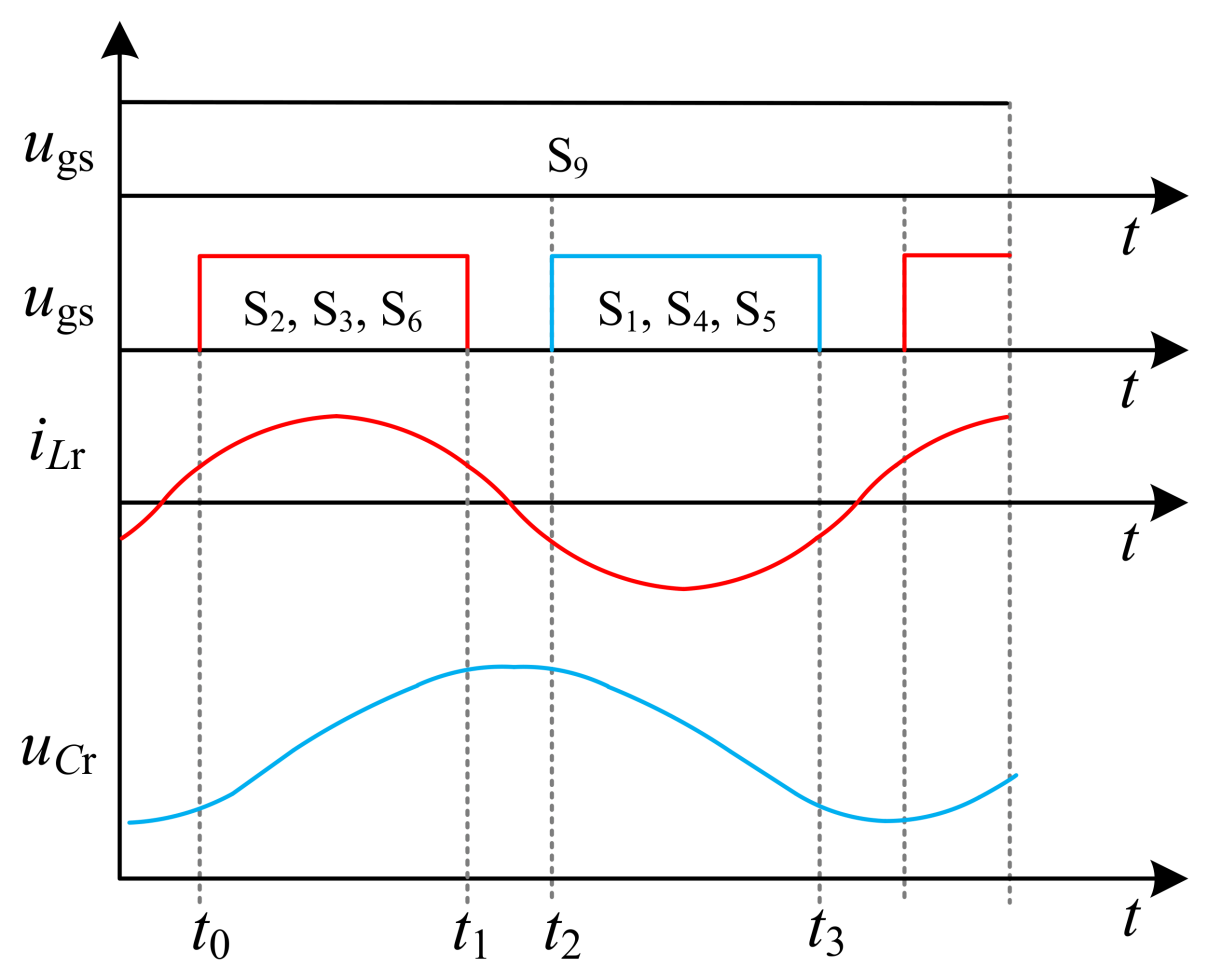
Main waveforms of CLG mode, where the intervals [*t*_0_–*t*_1_), [*t*_1_–*t*_2_), and [*t*_2_–*t*_3_] correspond to the positive half-cycle, negative half-cycle, and dead-time interval, respectively.

**Figure 7:**
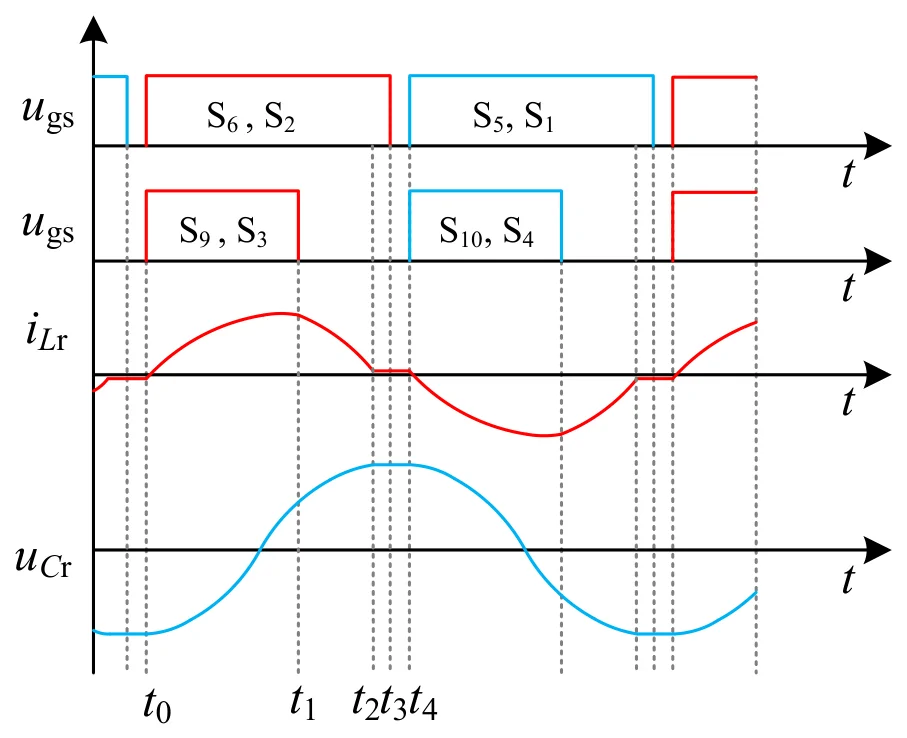
Main waveforms of CMG mode, where Stage 1, Stage 2, and Stage 3 correspond to the intervals [*t*_0_–*t*_1_), [*t*_1_–*t*_2_), and [*t*_2_–*t*_4_], respectively.

**Figure 8:**
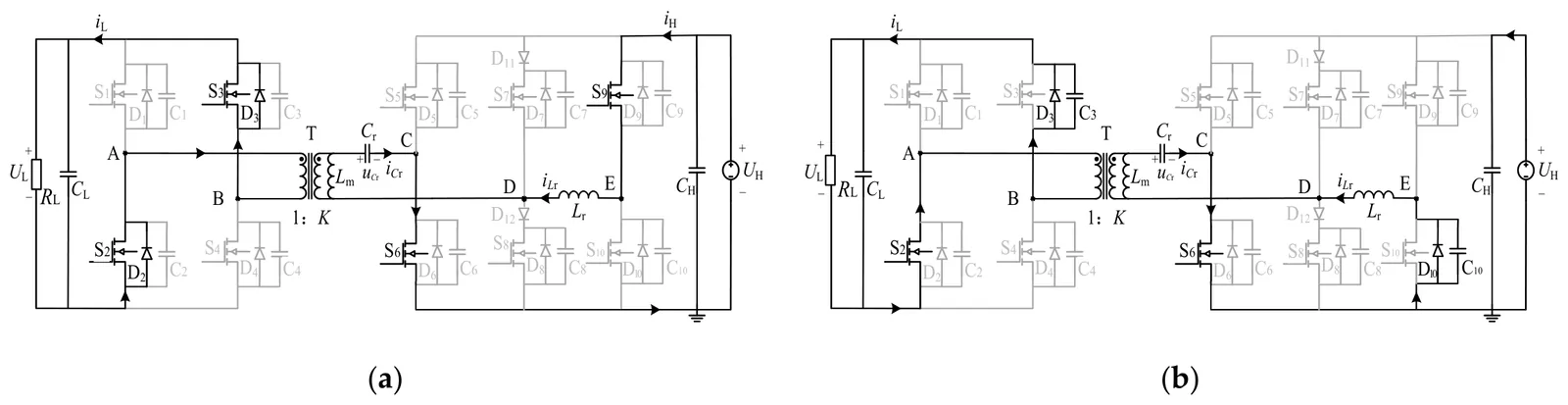
Equivalent circuits of each stage of CMG mode. (**a**) Stage 1. (**b**) Stage 2.

(1) Charging Low-Gain (CLG) Mode: When the battery-side voltage *U*_L_ is low, the converter operates in the CLG mode. On the HVS, only switches S_9_, S_5_, and S_6_ are active, while S_7_, S_8_, and S_10_ remain inactive. On the LVS, switches S_2_ and S_3_ operate synchronously with switch S_6_ on the HVS, while switches S_1_ and S_4_ operate synchronously with switch S_5_ on the HVS. Switch S_9_ remains on throughout the entire switching period. Switches S_1_–S_6_ operate with fixed duty cycles slightly lower than 0.5, and PFM is adopted. The main waveforms are shown in Figure 6. The operating principle of this mode is identical to that of the conventional half-bridge LLC resonant converter.

(2) Charging Medium-Gain (CMG) Mode: When *U*_L_ is at a medium level, the converter operates in the CMG mode with *f*_s_ = *f*_r_. The corresponding key waveforms are shown in Figure 7. Switches S_9_ and S_10_ are controlled by PWM, with a duty cycle of *D*_cm_ = 2(*t*_1_ − *t*_0_)/*T*_s_, while S_5_ and S_6_ operate with fixed duty cycles slightly lower than 0.5. On the LVS, switches S_1_, S_2_, S_3_, and S_4_ are driven synchronously with S_5_, S_6_, S_9_, and S_10_, respectively. The equivalent circuit is shown in Figure 8. The detailed operating process is described as follows.

Stage 1 [*t*_0_–*t*_1_): LC resonant stage. At *t*_0_, the initial value of *i_L_*_r_ is 0, and the resonant capacitor voltage *u_C_*_r_ is at its negative peak value, i.e., −Δ*U_C_*_rM_. Switches S_2_, S_3_, S_6_, and S_9_ are turned on under ZCS. Then, *L*_r_ and *C*_r_ start to resonate, and energy is transferred from the HVS to the LVS. During this stage, the time-domain expressions of *i_L_*_r_ and *u_C_*_r_ are given by

(4) $$\left\{\begin{array}{l} i_{Lr}(t)=\frac{U_{H}-KU_{L}+\Delta U_{CrM}}{Z_{r}}\sin[\omega_{r}(t-t_{0})]\\ u_{Cr}(t)=U_{H}-KU_{L}-(U_{H}-KU_{L}+\Delta U_{CrM})\cos[\omega_{r}(t-t_{0})]\; \end{array}\right.$$

Stage 2 [*t*_1_–*t*_2_): LC freewheeling stage. At *t*_1_, S_9_ is turned off, while S_6_ remains on. Since *i_L_*_r_ cannot change instantaneously, the body diode D_10_ of switch S_10_ becomes forward-biased, thereby providing a freewheeling path for the LC resonant current. During this stage, *i_L_*_r_ continuously decreases.

Stage 3 [*t*_2_–*t*_4_]: Turn-off and dead-time stage. At *t*_2_, *i_L_*_r_ decreases to 0. At *t*_3_, S_2_ and S_6_ are turned off under ZCS. During this stage, capacitor *C*_L_ supplies the load.

**Figure 9:**
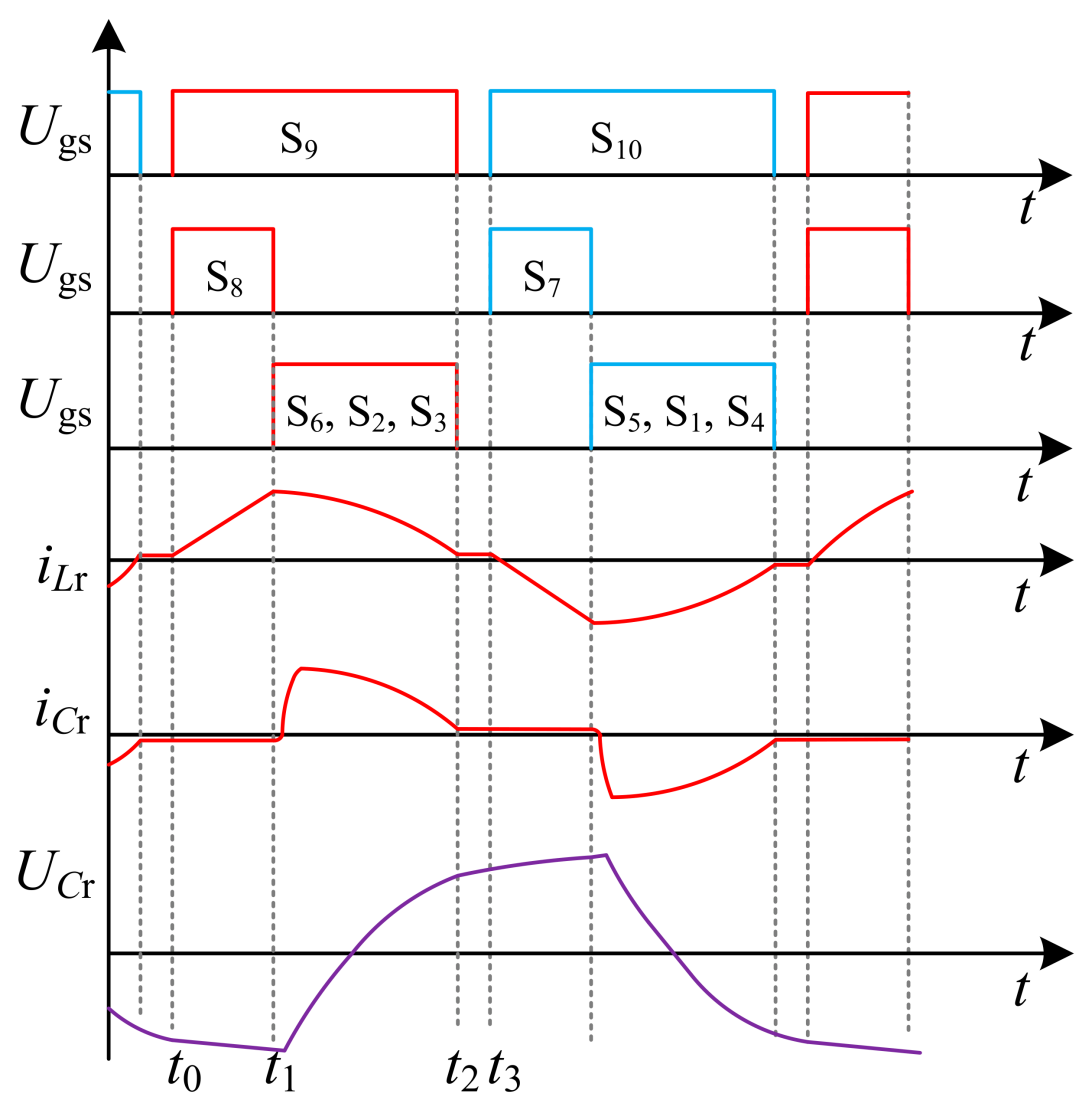
Main waveforms of CHG mode, where Stage 1, Stage 2, and Stage 3 correspond to the intervals [*t*_0_–*t*_1_), [*t*_1_–*t*_2_), and [*t*_2_–*t*_3_], respectively.

**Figure 10:**
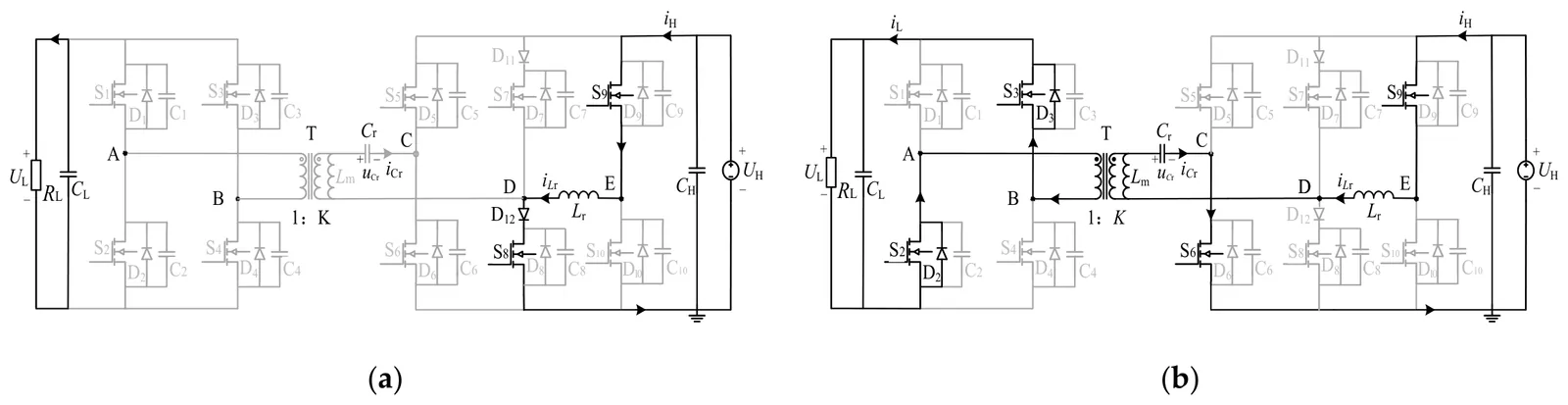
Equivalent circuits of each stage of CHG mode. (**a**) Stage 1. (**b**) Stage 2.

(3) Charging High-Gain (CHG) Mode: When *U*_L_ is high, the converter switches to the CHG mode with *f*_s_ = *f*_r_. The corresponding key waveforms are shown in Figure 9. On the HVS, all switches are involved in operation. S_7_ and S_8_ are controlled by PWM, with a duty cycle of *D*_ch_ = 2(*t*_1_–*t*_0_)/*T*_s_. S_9_ and S_10_ operate with a fixed duty cycle *D*_T_ slightly lower than 0.5, whereas S_5_ and S_6_ are PWM-controlled with a duty cycle of *D*_T_ − *D*_ch_. The corresponding equivalent circuits are shown in Figure 10. The detailed operating process is described as follows.

Stage 1 [*t*_0_–*t*_1_): Boost energy storage stage. At *t*_0_, S_9_ and S_8_ are turned on, and *U*_H_ charges *L*_r_, causing *i_L_*_r_ to increase linearly. At *t*_0_, *i_L_*_r_ can be expressed as

(5) $$i_{Lr}(t_{1})=\frac{U_{H}D_{\mathrm{ch}}}{2f_{s}L_{r}}$$

During this stage, energy is stored in *L*_r_, while on the LVS, capacitor *C*_L_ supplies energy to the load *R*_L_.

Stage 2 [*t*_1_–*t*_2_): LC resonant stage. At *t*_1_, S_8_ is turned off, and S_6_ is turned on immediately, while S_9_ remains on. At this instant, the capacitor voltage reaches its negative peak value, i.e., −Δ*U_C_*_rM_. Then, *U*_H_ excites the LC resonant tank, and the converter enters the LC resonant stage. During this stage, *u_C_*_r_ rapidly decreases to 0 and then reverses its polarity, while *i_L_*_r_ continuously decreases and approaches 0 at *t*_2_. The time-domain expressions of *i_L_*_r_ and *u_C_*_r_ in this stage are given by

(6) $$\left\{\begin{array}{l} i_{Lr}(t)=\frac{U_{H}D_{\mathrm{ch}}}{2f_{s}L_{r}}\cos[\omega_{r}(t-t_{1})]+\frac{U_{H}-KU_{L}+\Delta U_{C\mathrm{rM}}}{Z_{r}}\sin[\omega_{r}(t-t_{1})]\\ u_{Cr}(t)=U_{H}-KU_{L}+\omega_{r}i_{b}\sin[\omega_{r}(t-t_{1})]-(U_{H}-KU_{L}+\Delta U_{CrM})\cos[\omega_{r}(t-t_{1})] \end{array}\right.$$

Stage 3 [*t*_2_–*t*_3_]: dead-time stage. At *t*_2_, S_2_, S_3_, S_6_, and S_9_ are turned off under ZCS. During this short interval, capacitor *C*_L_ supplies the load *R*_L_.

The boost arm adopts a series-connected diode–MOSFET structure. This configuration can effectively reduce the turn-off loss of the boost arm and prevent reverse current flow.

## 3. Steady Gain Analysis

### 3.1. Voltage Gain G_DMG_ of DMG Mode

Based on the above analysis of the DMG mode, the output voltage gain of the DMG mode, denoted by *G*_DMG_, is defined as

(7) $$G_{\mathrm{DMG}}=\frac{U_{H}}{KU_{L}}$$

**Figure 11:**
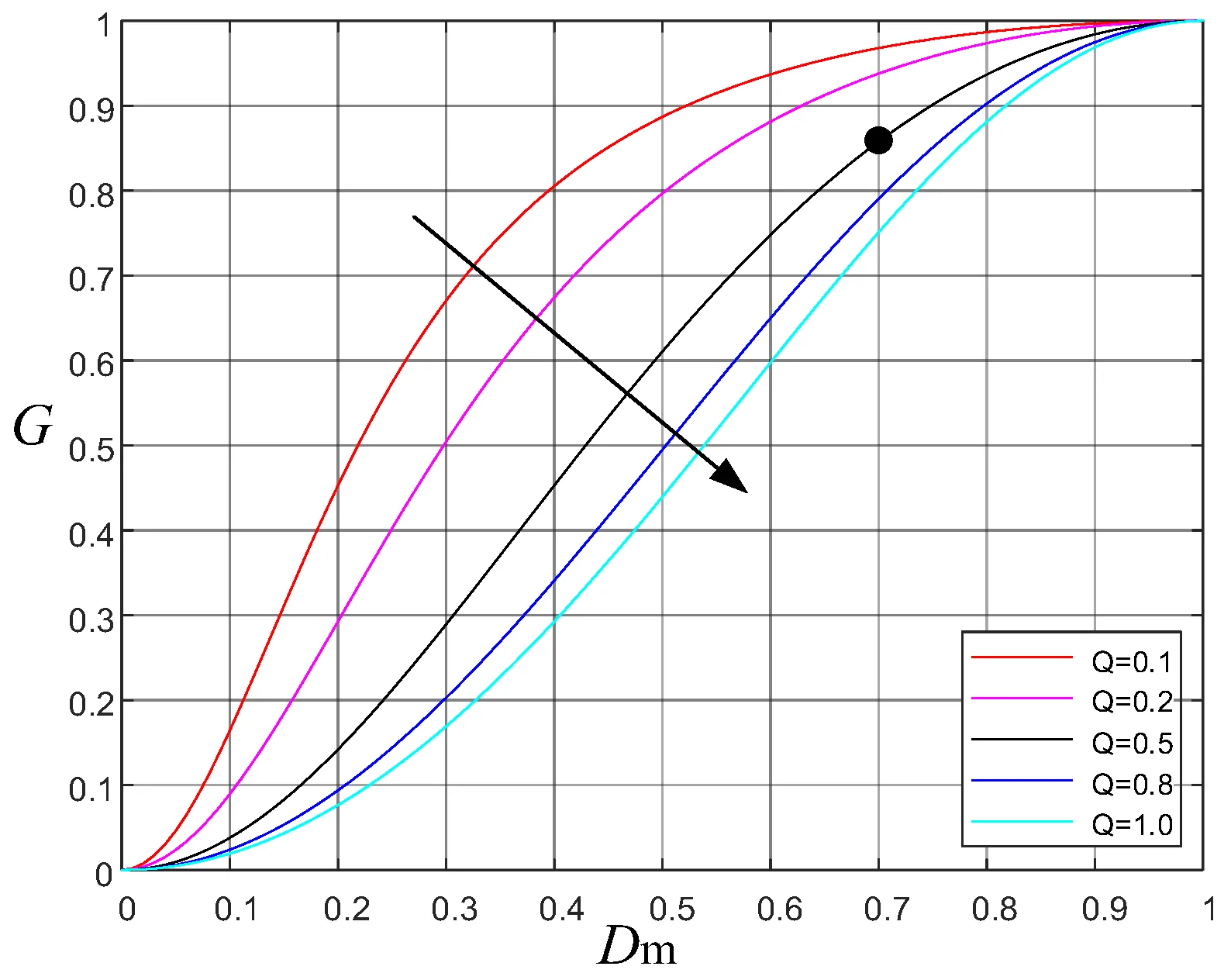
Voltage gain curves of DMG mode. The black marker indicates the selected design point used in the prototype, and the black arrow indicates the increasing direction of the quality factor *Q*.

After simplification, it can be expressed as

(8) $$G_{\mathrm{DMG}}=\frac{\pi \omega_{n}[1-\cos(\pi D_{m})]}{4Q[1+\cos(\pi D_{m})]+\pi \omega_{n}[1-\cos(\pi D_{m})]}$$

when *ω*_n_ = 1, the corresponding voltage gain characteristics are shown in Figure 11. It can be observed that, in the DMG mode, 0 ≤ *G*_DMG_ ≤ 1. Moreover, *G*_DMG_ increases with the increase in *D*_m_, whereas it decreases as the quality factor *Q* increases. In particular, when *D*_m_ lies in the intermediate range, *G*_DMG_ exhibits relatively strong sensitivity to variations in *Q*.

### 3.2. Voltage Gain G_DHG_ of DHG Mode

The DHG mode is essentially equivalent to the combination of *L*_r_ energy storage on the HVS and subsequent LC resonance. Therefore, its voltage gain is related to the duty cycle *D*_h_, which determines the duration of the *L*_r_ energy storage interval. The voltage gain of the DHG mode, denoted by *G*_DHG_, is defined in the same manner as in (7). After derivation, *G*_DHG_ can be expressed as

(9) $$G_{\mathrm{DHG}}=\frac{\sqrt{\pi Q}+\sqrt{-2\cos^{2}(\pi D_{h})+\pi Q+2}}{[1+\cos(\pi D_{h})]\sqrt{\pi Q}}$$

**Figure 12:**
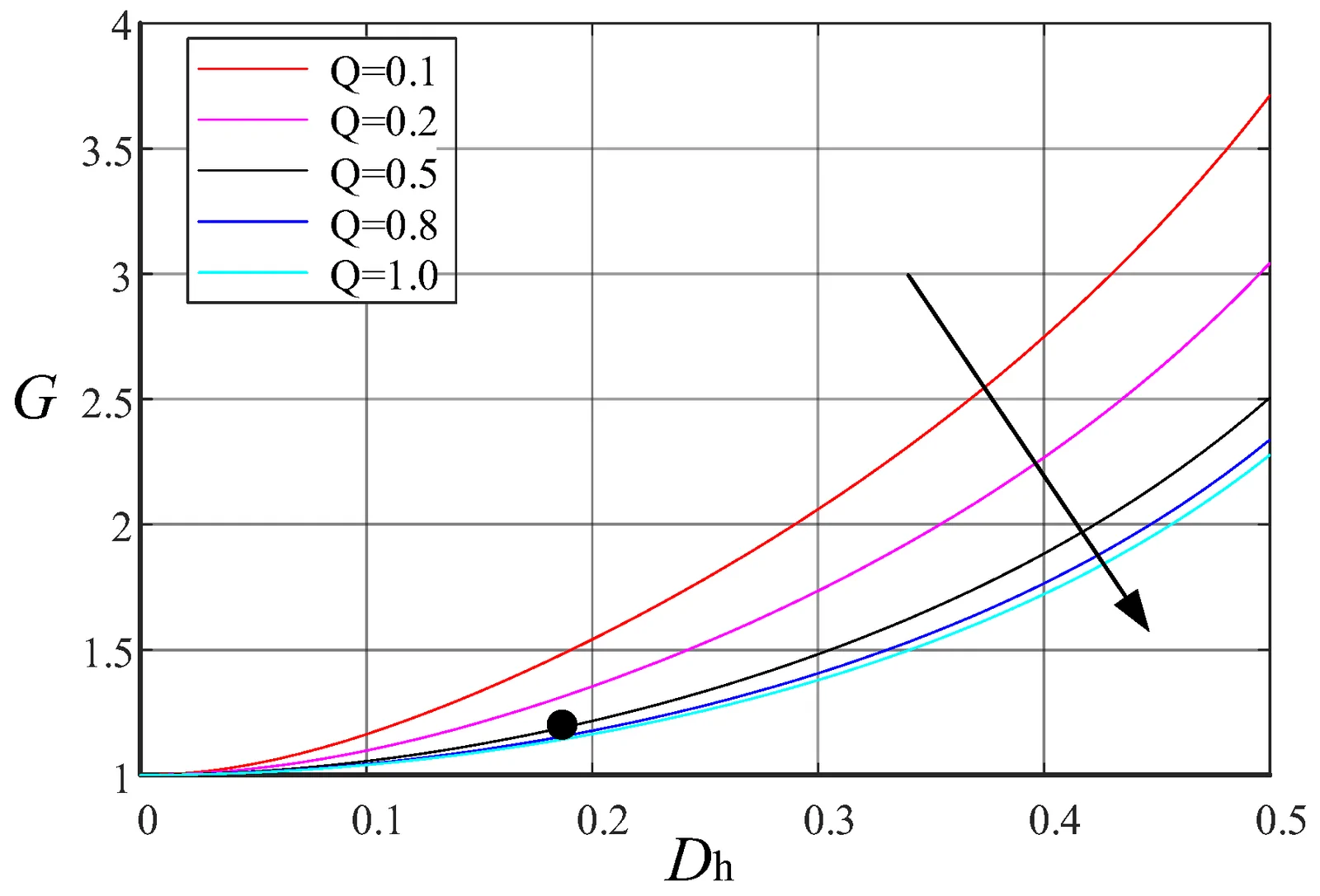
Voltage gain curves of DHG mode. The black marker indicates the selected design point used in the prototype, and the black arrow indicates the increasing direction of the quality factor *Q*.

The corresponding voltage gain characteristics are shown in Figure 12. It can be seen that in the DHG mode, *G*_DHG_ > 1. In addition, *G*_DHG_ increases with increasing *D*_h_, whereas it decreases as the quality factor *Q* increases for a given *D*_h_. Conversely, as *Q* increases, a larger *D*_h_ is required to maintain the same value of *G*_DHG_.

### 3.3. Voltage Gain G_CLG_ of CLG Mode

The CLG mode operates under PFM. The voltage gain of the CLG mode, denoted by *G*_CLG_, is defined as

(10) $$G_{\mathrm{CLG}}=KU_{L}/U_{H}$$

After calculation, *G*_CLG_ can be expressed as

(11) $$G_{\mathrm{CLG}}=\frac{1}{2\sqrt{1+Q^{2}{\left(\omega_{n}-1/\omega_{n}\right)}^{2}}}$$

**Figure 13:**
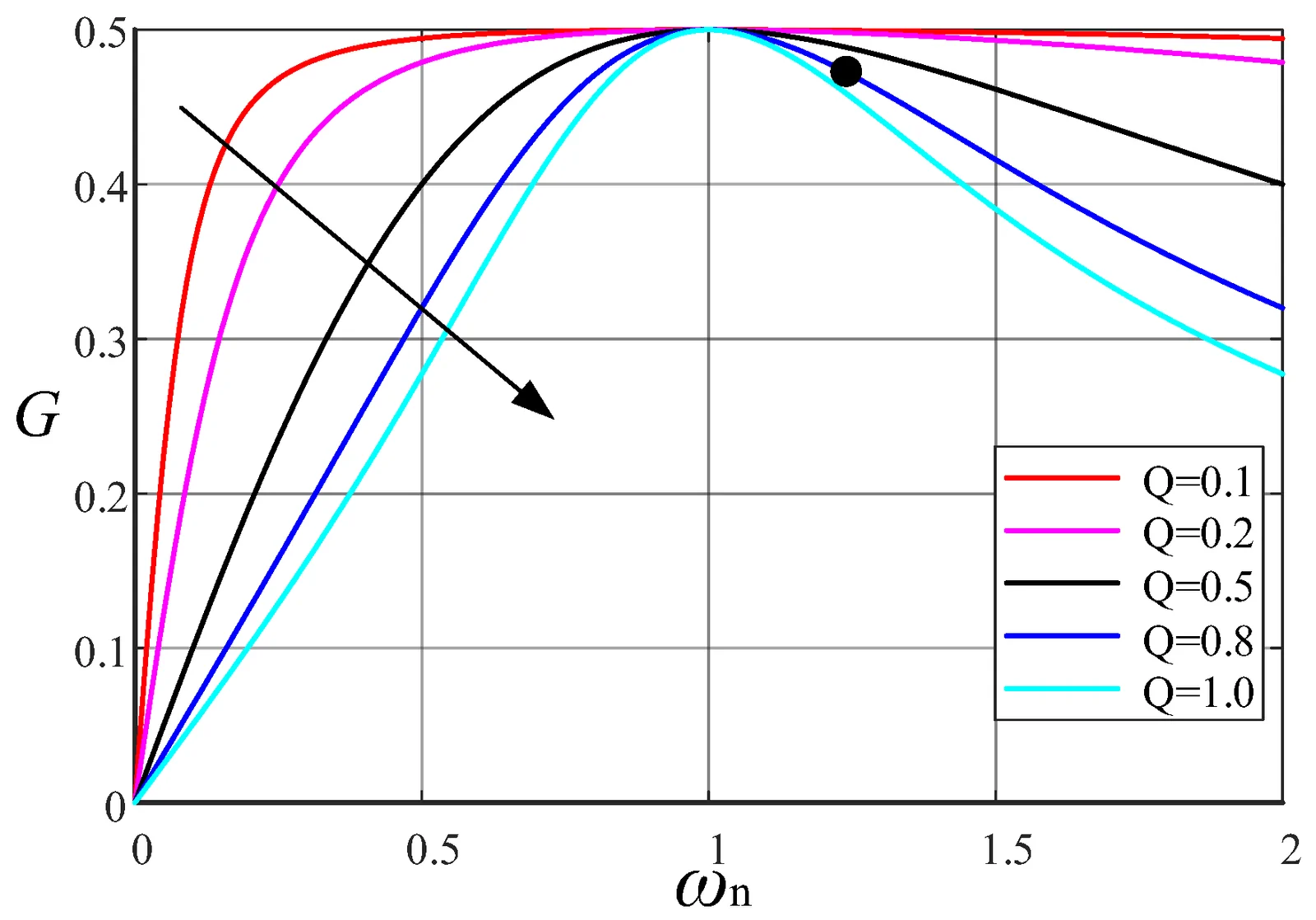
Voltage gain curves of CLG mode. The black marker indicates the selected design point used in the prototype, and the black arrow indicates the increasing direction of the quality factor *Q*.

The corresponding voltage gain characteristics are shown in Figure 13. It can be observed that *G*_CLG_ reaches its maximum at *f*_n_ = 1. As the operating frequency deviates further from *f*_n_, *G*_CLG_ decreases. In addition, an increase in the quality factor *Q* also leads to a reduction in *G*_CLG_.

### 3.4. Voltage Gain G_CMG_ of CMG Mode

This mode is essentially a fixed-frequency phase-shift-controlled LLC converter. Therefore, its voltage gain *G*_CMG_ has the same expression as that of the DMG mode. The gain characteristics of the CMG mode can be obtained directly by replacing *D*_m_ in the DMG mode expression with the phase-shift duty cycle *D*_cm_ and are, thus, identical to those of *G*_DMG_.

### 3.5. Voltage Gain G_CHG_ of CHG Mode

The CHG mode can be regarded as a combination of a Boost converter on the HVS and an LC resonant converter. Therefore, its voltage gain is related to the duty cycle *D*_ch_, which determines the energy storage interval of the Boost operation. The derivation is given as follows.

During the positive half-cycle of *i_Lr_*, the average value of *i_Lr_* is equal to the average input current on the HVS, i.e., *I*_H_, which can be expressed as

(12) $$I_{H}=2f_{s}\int_{t_{0}}^{t_{2}}i_{Lr}(t)dt=2f_{s}\int_{t_{0}}^{t_{1}}i_{Lr}(t)dt+2f_{s}\int_{t_{1}}^{t_{2}}i_{Lr}(t)dt=\frac{D_{\mathrm{ch}}^{2}U_{H}}{4f_{s}L_{r}}+\frac{I_{L}}{K}$$

Neglecting power losses, the average input current on the LVS, *I*_L_, can also be expressed as

(13) $$I_{L}=\frac{P_{\mathrm{in}}}{U_{H}}=\frac{P_{o}}{U_{H}}=\frac{U_{L}^{2}}{U_{H}R_{L}}$$

where *P*_in_ is the input power on the LVS, and *P*_o_ is the output power on the HVS.

By combining (12) and (13), the following expression can be obtained:

(14) $$G_{\mathrm{CHG}}=\frac{1}{2}+\frac{1}{4}\sqrt{4+(D_{\mathrm{ch}}^{2}\pi^{3})/(Qf_{n})}$$

**Figure 14:**
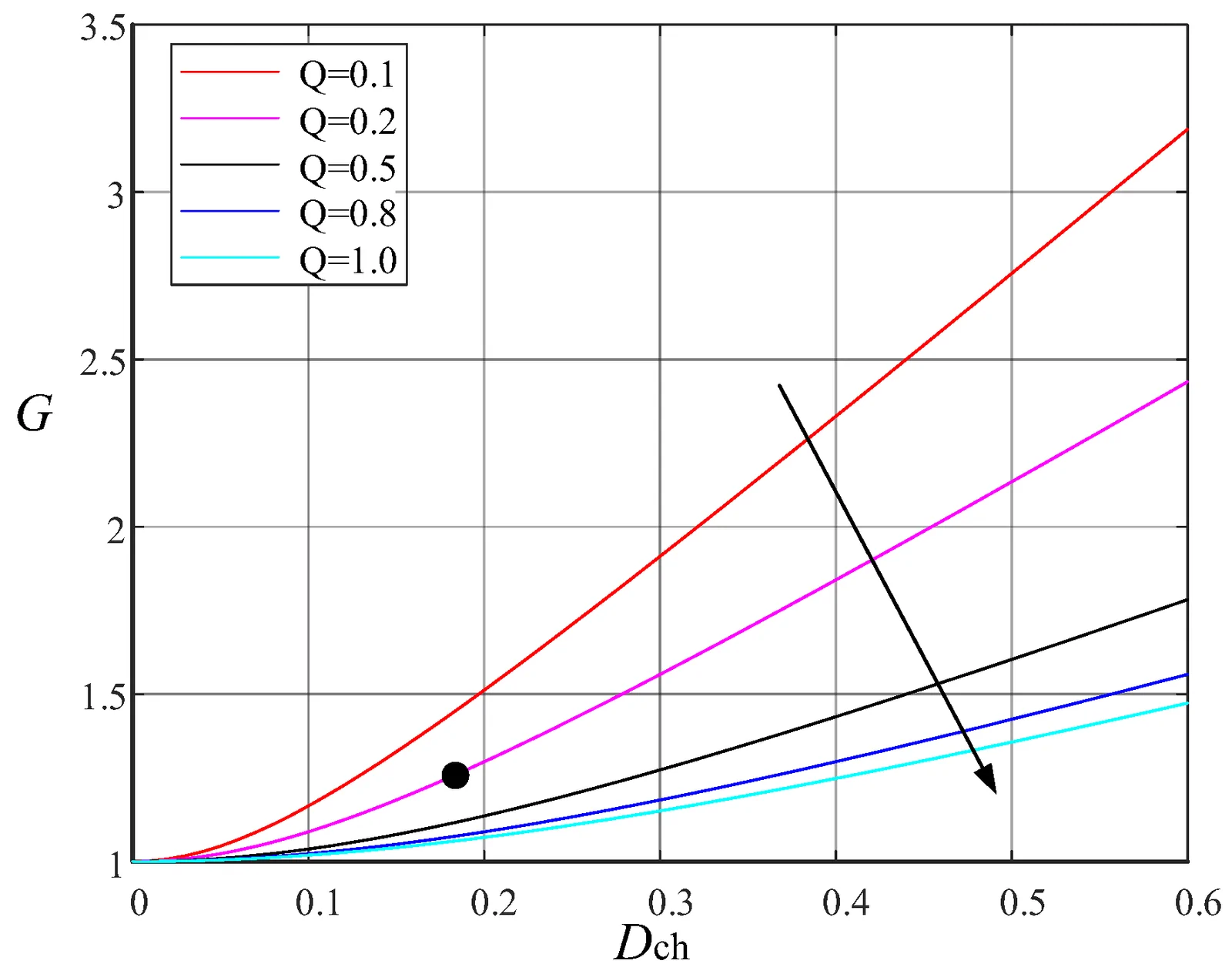
Gain curve of CHG mode. The black marker indicates the selected design point used in the prototype, and the black arrow indicates the increasing direction of the quality factor Q.

According to (14), by setting *f*_n_ = 1, the corresponding voltage-gain characteristics can be obtained, as shown in Figure 14. It can be seen that as *D*_ch_ increases, the maximum value of *G*_CHG_ also increases continuously. Moreover, the converter is able to maintain favorable linear regulation capability even under relatively large values of *Q*.

To further validate the derived voltage-gain expressions, the calculated and measured gains at representative experimental operating points are compared in Table 1. As shown in the table, the calculated gains are generally consistent with the measured results.

## 4. Design Guidelines

A bidirectional DC–DC converter was designed in this study for charging and discharging battery packs consisting of 7–10 cells with a rated voltage of 12 V per cell. The discharging voltage range is 40–120 V, and the charging voltage range is also 40–120 V. The detailed design parameters are listed in Table 2.

### 4.1. Forward and Reverse Voltage Gains

Assume that the maximum required discharging gain and charging gain of the bidirectional DC–DC converter are *G*_F_ and *G*_R_, respectively. When the turns ratio of the main transformer is *K*_1_ = 1, let the maximum forward gain and maximum reverse gain achievable by the bidirectional DC–DC converter be *G*_1_ and *G*_2_, respectively. Then, the transformer turns ratio *K* of the designed bidirectional DC–DC converter should satisfy

(15) $$\left\{\begin{array}{l} \frac{1}{K}G_{1}\geq G_{F}\\ KG_{2}\geq G_{R} \end{array}\right.$$

In order for the converter to meet the practical gain requirements, *K* must have a feasible solution. Therefore, the converter gain should satisfy

(16) $$G_{F}G_{R}\leq G_{1}G_{2}$$

According to Table 2, *G*_F_ = 4 and *G*_R_ = 0.65. Substituting these values into (16) yields *G*_1_*G*_2_ ≥ 2.6.

As can be seen from Figure 12 and Figure 14, the proposed converter can readily satisfy this bidirectional gain requirement by properly selecting the circuit parameters. Here, *G*_CHG_ = *G*_1_ = 2.05 and *G*_DHG_ = *G*_2_ = 1.35 are selected.

### 4.2. Transformer Turns Ratio Design

After determining *G*_CHG_ and *G*_DHG_, substituting them into (15) gives 1.95 ≤ *K* ≤ 2.07. For ease of prototype implementation, *K* is designed as 2.

By defining 0.7*G*_CHG_ as the rated gain *G*_o_, the converter operates in the medium-gain mode when the actual operating gain is lower than *G*_o_ and switches to the high-gain mode when the gain exceeds *G*_o_. The quality factor at the rated gain is defined as *Q*_o_ = 0.4.

### 4.3. Resonant Parameter Design

According to *f*_r_ and *Q*_o_, *L*_r_ and *C*_r_ can be designed as

(17) $$\left\{\begin{array}{l} L_{r}=\frac{8U_{L}^{2}G_{o}^{2}Q_{o}}{\pi^{2}\omega_{s}P_{i}}\\ C_{r}=\frac{\pi^{2}P_{i}}{8U_{L}^{2}G_{o}^{2}\omega_{s}Q_{o}} \end{array}\right.$$

where *P*_i_ is the rated power, and $\omega_{s}=2\pi f_{s}$ is the angular frequency.

Substituting the corresponding parameters into (17) yields *L*_r_ = 15.8 μH and *C*_r_ = 160 nF.

### 4.4. Control Strategy

**Figure 15:**
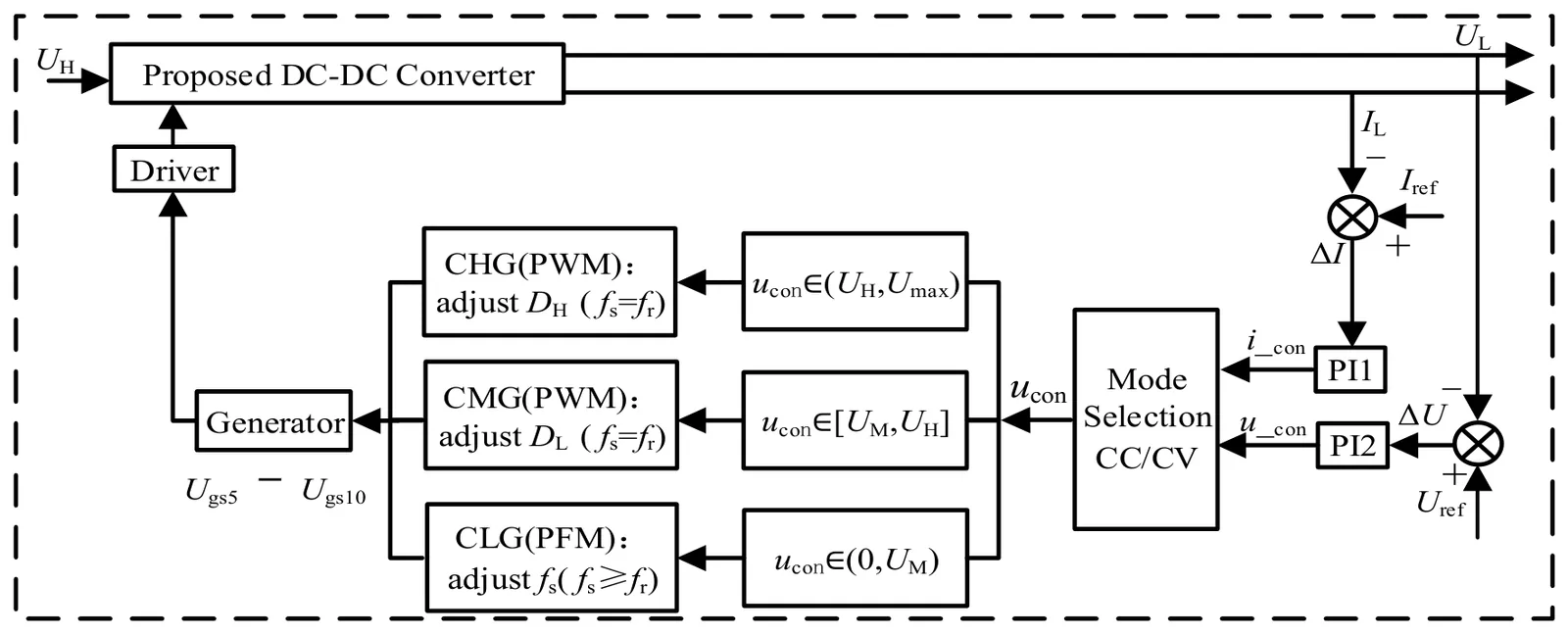
Control block diagram of converter.

The proposed converter achieves stable output regulation by means of a dual-loop control scheme consisting of an inner current loop and an outer voltage loop. Taking the charging mode as an example, the corresponding control block diagram is shown in Figure 15.

The LVS voltage *U*_L_ and current *I*_L_ are sampled in real time and compared with their reference values *U*_ref_ and *I*_ref_, respectively. Here, *u*__con_ represents the output of the voltage-loop PI controller, which is used as the mode selection variable. *i*__con_ denotes the output of the current-loop PI controller. *U*_H_, *U*_M_, and *U*_max_ are the predefined control thresholds for charging-mode transition, where *U*_H_ and *U*_M_ represent the boundaries between CHG, CMG, and CLG modes, respectively, and *U*_max_ represents the maximum control limit. The resulting error signals are processed by proportional–integral (PI) controllers to generate the voltage control signal *u*__con_ and the current control signal *i*__con_. Through mode selection, the final control signal *u*_con_ is obtained. When *U*_L_ approaches *U*_ref_, the controller switches to voltage loop regulation.

The operating ranges of the three charging modes are defined as follows: (1) CHG mode, *u*_con_∈ (*U*_H_, *U*_max_); (2) CMG mode, *u*_con_∈ [*U*_M_, *U*_H_]; and (3) CLG mode, *u*_con_∈ (0, *U*_M_), where *U*_H_ and *U*_M_ are the control-voltage thresholds corresponding to the charging-mode transition boundaries. When *u*_con_ ≥ *U*_M_, the converter operates in PWM mode at the resonant frequency *f*_r_, and the output voltage is regulated by adjusting the duty cycle *D*. When *u*_con_ < *U*_M_, the converter operates in PFM mode, and the output is controlled by adjusting the switching frequency *f*_s_. The control strategy in the discharging mode is similar.

The same PI parameters are used in all operating modes. In the experimental prototype, the current-loop PI1 parameters are *K*_pi_ = 16 and *K*_ii_ = 0.0001, while the voltage-loop PI2 parameters are *K*_pv_ = 0.003 and *K*_iv_ = 0.0001.

## 5. Experimental Results

**Figure 16:**
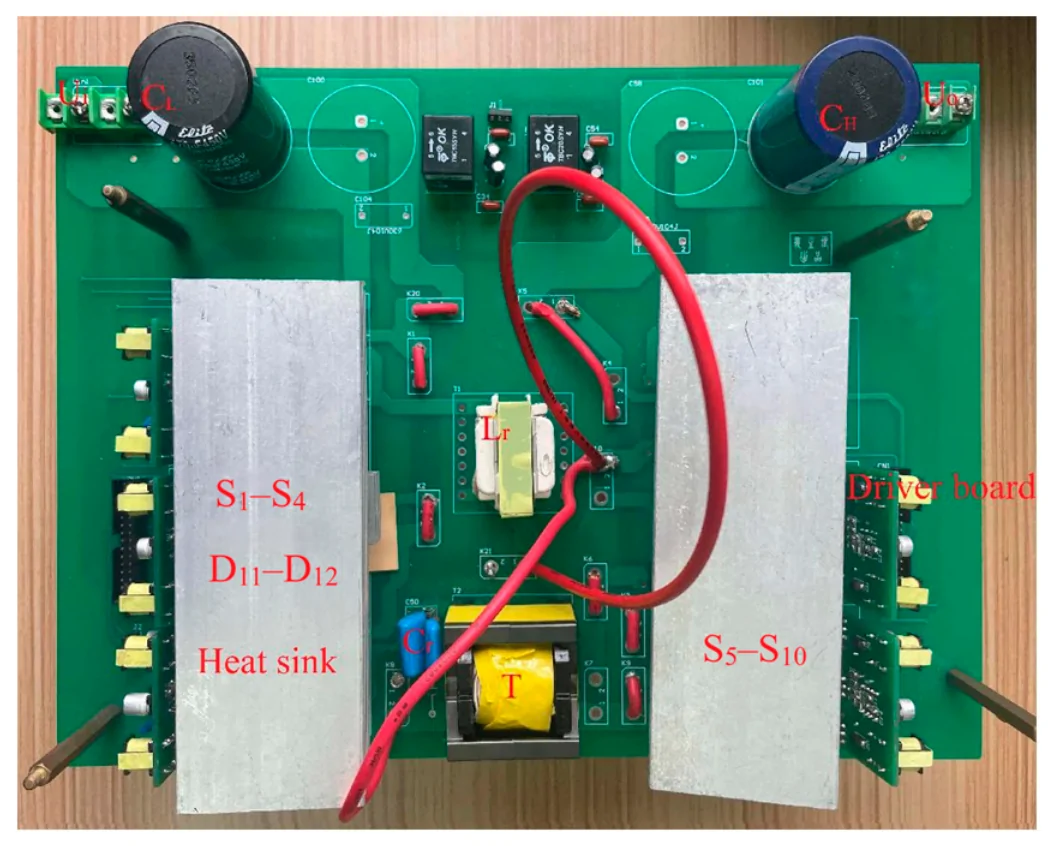
Photo of the experimental prototype.

To verify the proposed bidirectional DC–DC converter, a laboratory prototype was built and tested. The prototype has a rated charging/discharging power of 750 W, a rated bus-side voltage of 200 V, and a rated battery-side voltage of 94 V, with an operating range of 40–120 V. The switching frequency is 100 kHz. For transformer T, the inductance on the high-voltage side is 300 μH with a leakage inductance of 2.6 μH, while the inductance on the low-voltage side is 75 μH with a leakage inductance of 0.6 μH. A photograph of the prototype is shown in Figure 16. The operating parameters of the prototype and the main component parameters are listed in Table 3.

### 5.1. Experimental Waveforms in Discharging Operation

The designed bidirectional DC–DC converter operates over a discharging range with an input voltage of 40–120 V and an output voltage of 200 V.

**Figure 17:**
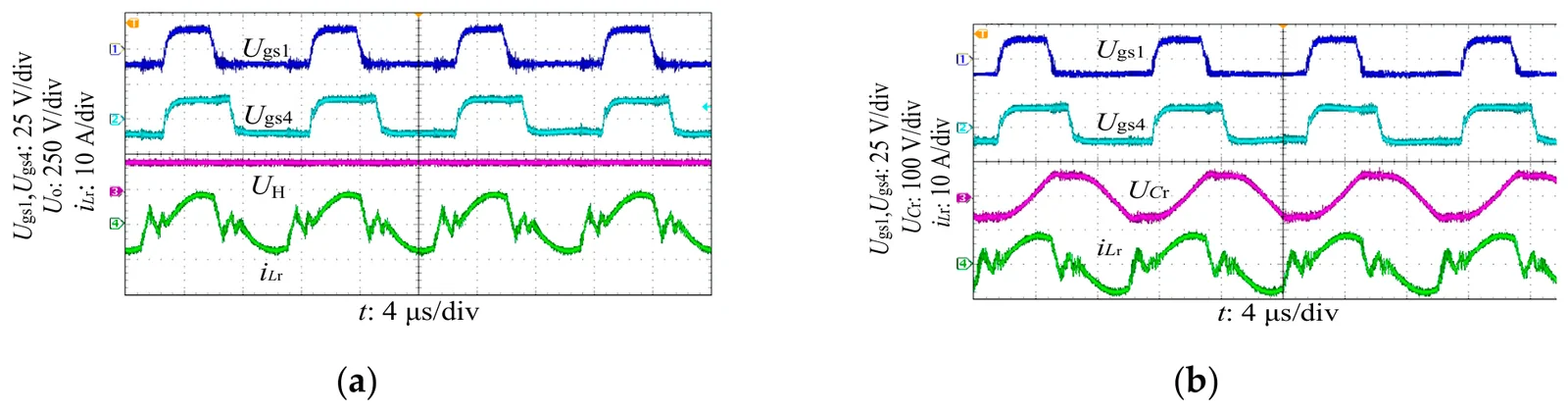
Key waveforms with 200 V output in DMG mode under *U*_L_ = 115 V, *U*_H_ = 200 V, *D*_m_ = 0.7, and *R*_H_ = 51 Ω. (**a**) Experimental waveforms of *U*_gs1_, *U*_gs4_, *i*_Lr_, and *U*_H_. (**b**) Experimental waveforms of *U*_gs1_, *U*_gs4_, *i*_Lr_, and *U*_Cr_.

**Figure 18:**
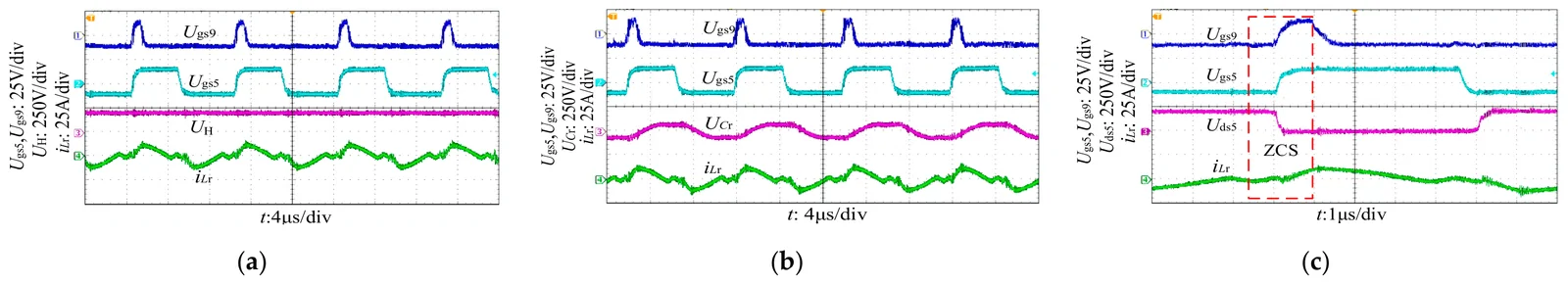
Key waveforms with 200 V output in DHG mode under *U*_L_ = 84 V, *U*_H_ = 200 V, *D*_h_ = 0.18, and *R*_H_ = 53.8 Ω. (**a**) Experimental waveforms of *U*_gs5_, *U*_gs9_, *U*_H_, and *i*_Lr_. (**b**) Experimental waveforms of *U*_gs5_, *U*_gs9_, *U_C_*_r_, and *i*_Lr_. (**c**) Soft-switching waveforms.

Figure 17 shows the key waveforms in the DMG mode when the LVS voltage is *U*_L_ = 115 V, the duty cycle is *D*_m_ = 0.7, and the load resistance is *R*_H_ = 51 Ω, corresponding to a HVS output of *U*_H_ = 200 V. As can be seen from Figure 18, when switches S_1_ and S_4_ are turned on, the inductor and capacitor resonate, and the current exhibits a sinusoidal waveform. After S_1_ is turned off, the current decreases rapidly. The initial current at the turn-on instants of S_1_ and S_4_ is approximately 0, indicating that zero-current turn-on is achieved. In addition, the voltage across S_4_ is 0 at its turn-off instant, thereby achieving zero-current turn-off.

Figure 18 shows the key waveforms in the DHG mode when the LVS voltage is *U*_L_ = 84 V, the duty cycle is *D*_h_ = 0.18, and the load resistance is *R*_H_ = 53.8 Ω, corresponding to a HVS output of *U*_H_ = 200 V. It can be observed from Figure 18 that when switches S_1_, S_4_, S_5_, and S_9_ are turned on, the inductor stores energy linearly. After S_9_ is turned off, diode D_10_ becomes forward-biased, and the converter enters the resonant stage. As shown in Figure 18c, the initial current at the turn-on instants of S_5_ and S_9_ is approximately 0, which confirms that zero-current turn-on is achieved. Moreover, the voltage across S_5_ is 0 at its turn-off instant, indicating zero-current turn-off.

### 5.2. Experimental Waveforms in Charging Operation

In the charging operation of the designed bidirectional DC–DC converter, the input voltage on the HVS is 200 V, and the output voltage on the LVS ranges from 40 V to 120 V.

**Figure 19:**
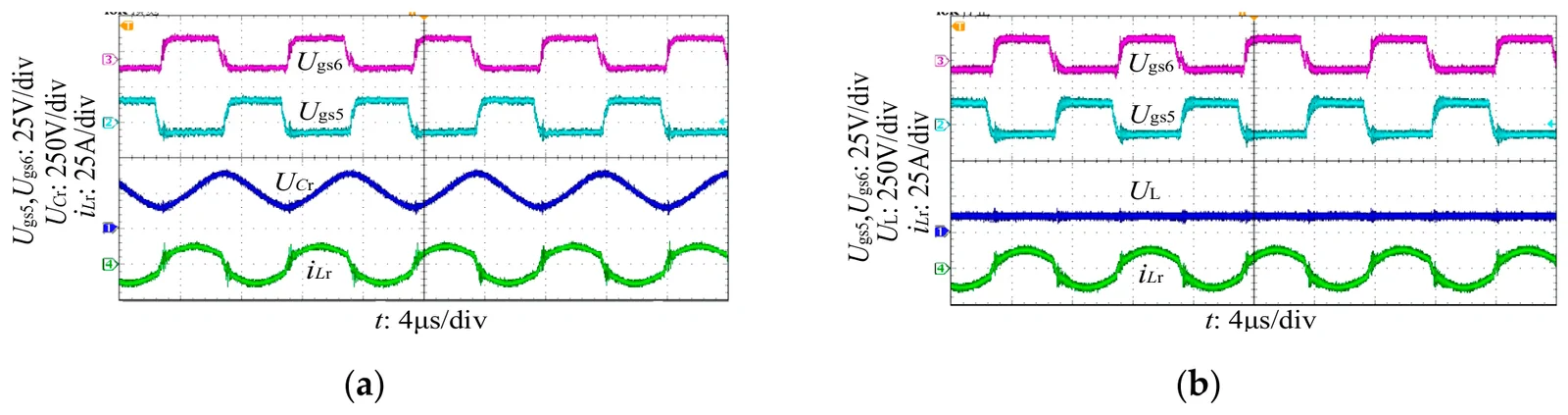
Key waveforms in CLG mode under *U*_L_ = 46 V, *U*_H_ = 200 V, *f*_s_ = 120 kHz, and *R*_L_ = 6.6 Ω. (**a**) Experimental waveforms of *U*_gs5_, *U*_gs6_, *U_C_*_r_, and *i*_Lr_. (**b**) Experimental waveforms of *U*_gs5_, *U*_gs6_, *U*_L_, and *i_L_*_r_.

In the CLG mode, the key waveforms under the conditions of *U*_H_ = 200 V, *f*_s_ = 120 kHz, *R*_L_ = 6.6 Ω, and *U*_L_ = 46 V are shown in Figure 19. It can be seen that when S_5_ and S_6_ are turned off, a certain conduction current still exists in the switches, indicating the presence of circulating power loss in the converter.

**Figure 20:**
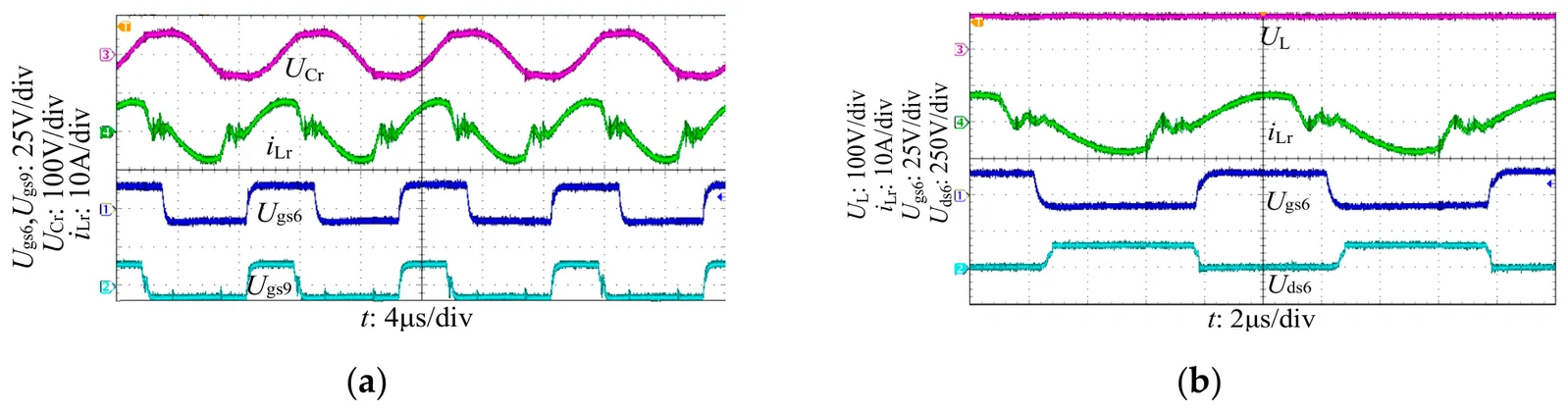
Key waveforms in CMG mode under *U*_L_ = 84 V, *U*_H_ = 200 V, *D*_cm_ = 0.8, and *R*_L_ = 9.8 Ω. (**a**) Experimental waveforms of *U*_gs6_, *U*_gs9_, *U_C_*_r_, and *i*_Lr_. (**b**) Experimental waveforms of *U*_gs6_, *U*_ds6_, *U*_L_, and *i*_Lr_.

In the CMG mode, the key waveforms under the conditions of *U*_H_ = 200 V, *D*_cm_ = 0.8, *R*_L_ = 9.8 Ω, and *U*_L_ = 84 V are shown in Figure 20. When S_6_ and S_9_ are turned on, *L*_r_ and *C*_r_ resonate. The soft-switching principle is the same as that in the DMG mode. Specifically, S_6_ and S_9_ achieve ZCS turn-on, and S_6_ also achieves ZCS turn-off.

**Figure 21:**
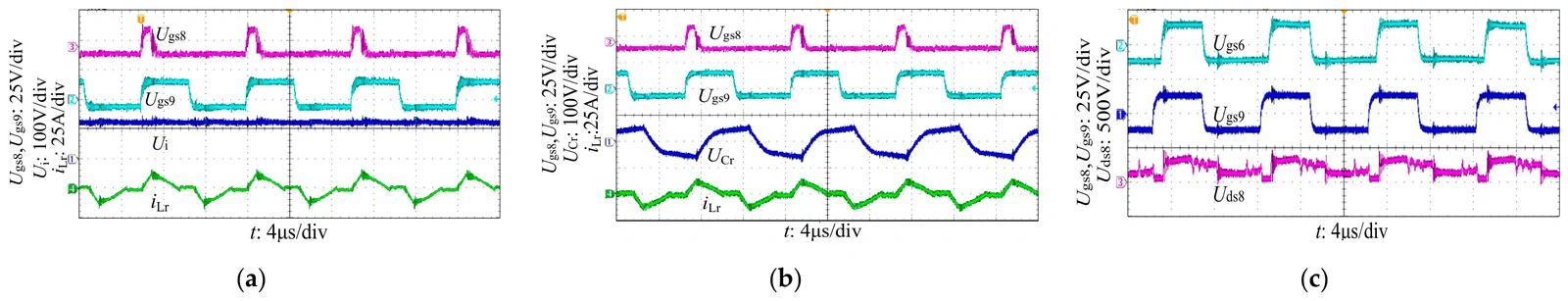
Key waveforms in CHG mode under *U*_L_ = 127 V, *U*_H_ = 200 V, *D*_ch_ = 0.18, and *R*_L_ = 20 Ω. (**a**) Experimental waveforms of *U*_gs8_, *U*_gs9_, *U*_L_, and *i*_Lr_. (**b**) Experimental waveforms of *U*_gs8_, *U*_gs9_, *U_C_*_r_, and *i*_Lr_. (**c**) Experimental waveforms of *U*_gs6_, *U*_gs9_, and *U*_ds8_.

In the CHG mode, the key waveforms under the conditions of *U*_H_ = 200 V, *D*_ch_ = 0.18, *R*_L_ = 20 Ω, and *U*_L_ = 127 V are shown in Figure 21. It can be observed that when S_8_ and S_9_ are turned on, the current through *L*_r_ increases linearly. After S_8_ is turned off, S_6_ is turned on, and the converter enters the resonant stage. During this process, S_8_ and S_9_ achieve zero-current turn-on, while S_6_ and S_9_ achieve zero-current turn-off. As can be seen from Figure 21c, the turn-off voltage stress of switch S_8_ (or S_7_) in the boost arm is 470 V. The detailed comparison of the key component voltage and current stresses in CHG and DHG modes is provided in Table S1 of the Supplementary Materials.

**Figure 22:**
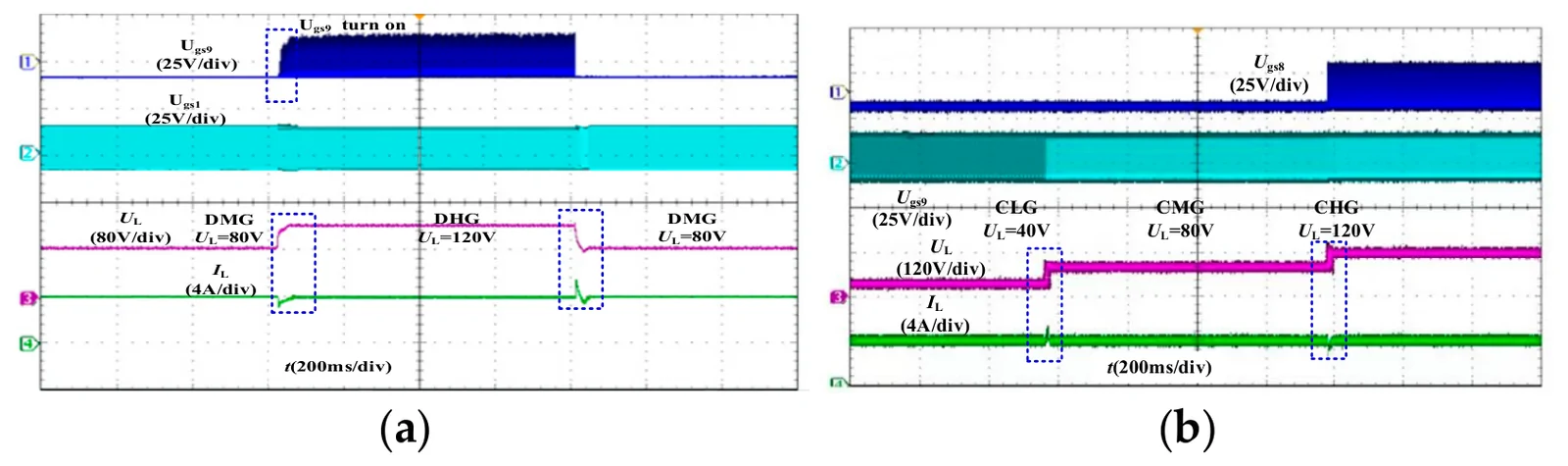
Experimental waveforms during dynamic mode transitions. (**a**) Experimental waveform of transition from DMG mode to DHG mode. (**b**) Experimental waveform of transitions among CLG, CMG, and CHG modes.The dashed blue boxes indicate the variations of U_L_ and *I*_L_ during the switching process.

Figure 22 shows the experimental waveforms of the dynamic transitions between different operating modes. In Figure 22a, the converter transitions between the DMG and DHG modes as the battery-side voltage *U*_L_ is adjusted. No noticeable voltage overshoot is observed, while a brief current pulse occurs, and the settling time is about 30 ms. Figure 22b presents the charging-mode transitions from CLG to CMG and then to CHG. The voltage overshoot is approximately 10%, the transient current pulse is about 15% of the normal operating current, and the settling time is approximately 10 ms. The results indicate that stable mode transitions can be achieved without excessive voltage or current stress.

### 5.3. Efficiency

**Figure 23:**
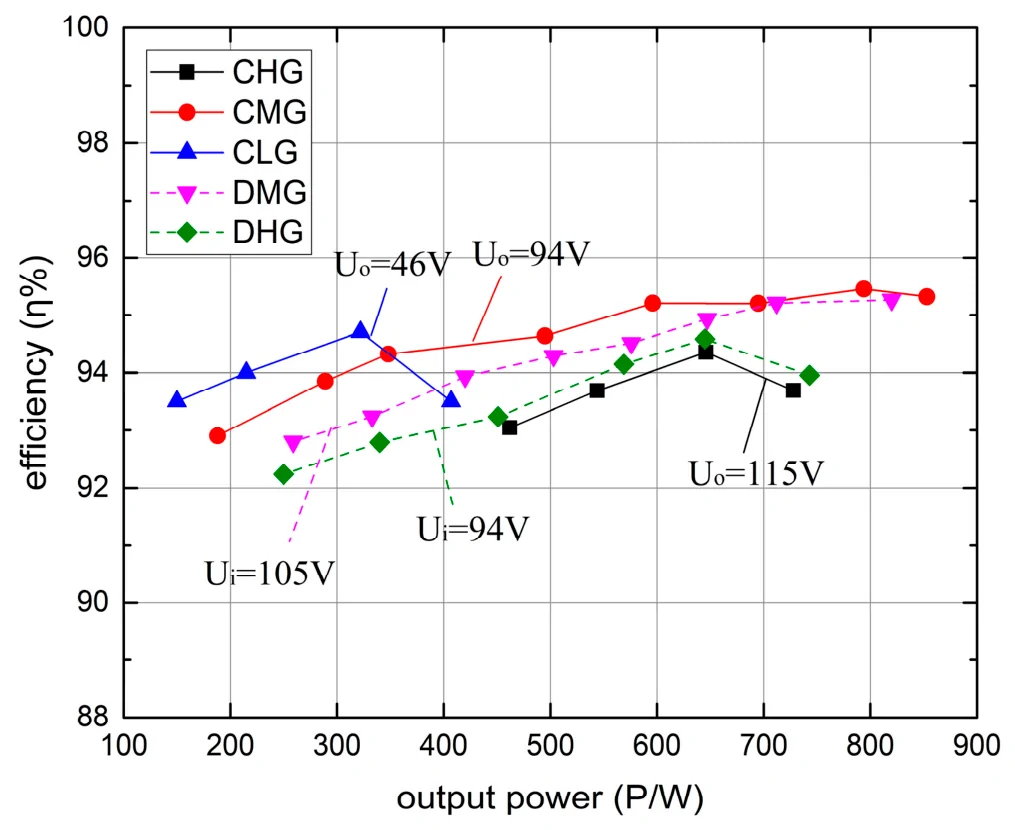
Efficiency curves under different modes.

Figure 23 shows the efficiency curves of the bidirectional DC–DC converter under different operating modes during both charging and discharging. The measured efficiency excludes the gate-driver, control-board, and auxiliary-supply losses and was recorded under steady-state operation. The test conditions are as follows: the high-voltage-side input voltage is 200 V during charging, whereas the high-voltage-side output voltage is 200 V during discharging. Representative numerical efficiency values corresponding to the curves in Figure 23 are summarized in Table 4.

It can be observed that, in the discharging mode, when the low-voltage-side battery voltage is 105 V, the maximum efficiency reaches 95.3% at rated power. When the output power is 300 W, the efficiency is 93.0%. In general, the farther the operating point deviates from the rated power, the lower the efficiency becomes. In the DHG mode, when the low-voltage-side battery voltage is 94 V, and the peak efficiency reaches 94.6% near rated power, whereas the efficiency drops to 92.5% when the output power is 300 W.

During charging operation, the overall efficiencies of the CMG and CLG modes are higher than those of the CHG mode. This is because the CMG and CLG modes operate only in the resonant state, and the switches work under soft-switching conditions, resulting in higher efficiency. In the CMG mode, the efficiency can reach 95.4% under rated output voltage and rated power. The CLG mode is suitable for applications with *U*_L_ ≤ 48 V and relatively light loads, while its efficiency decreases rapidly when the load power exceeds 350 W. The CHG mode is suitable for applications with *U*_L_ ≥ 96 V, and its maximum efficiency reaches 94.4%.

**Figure 24:**
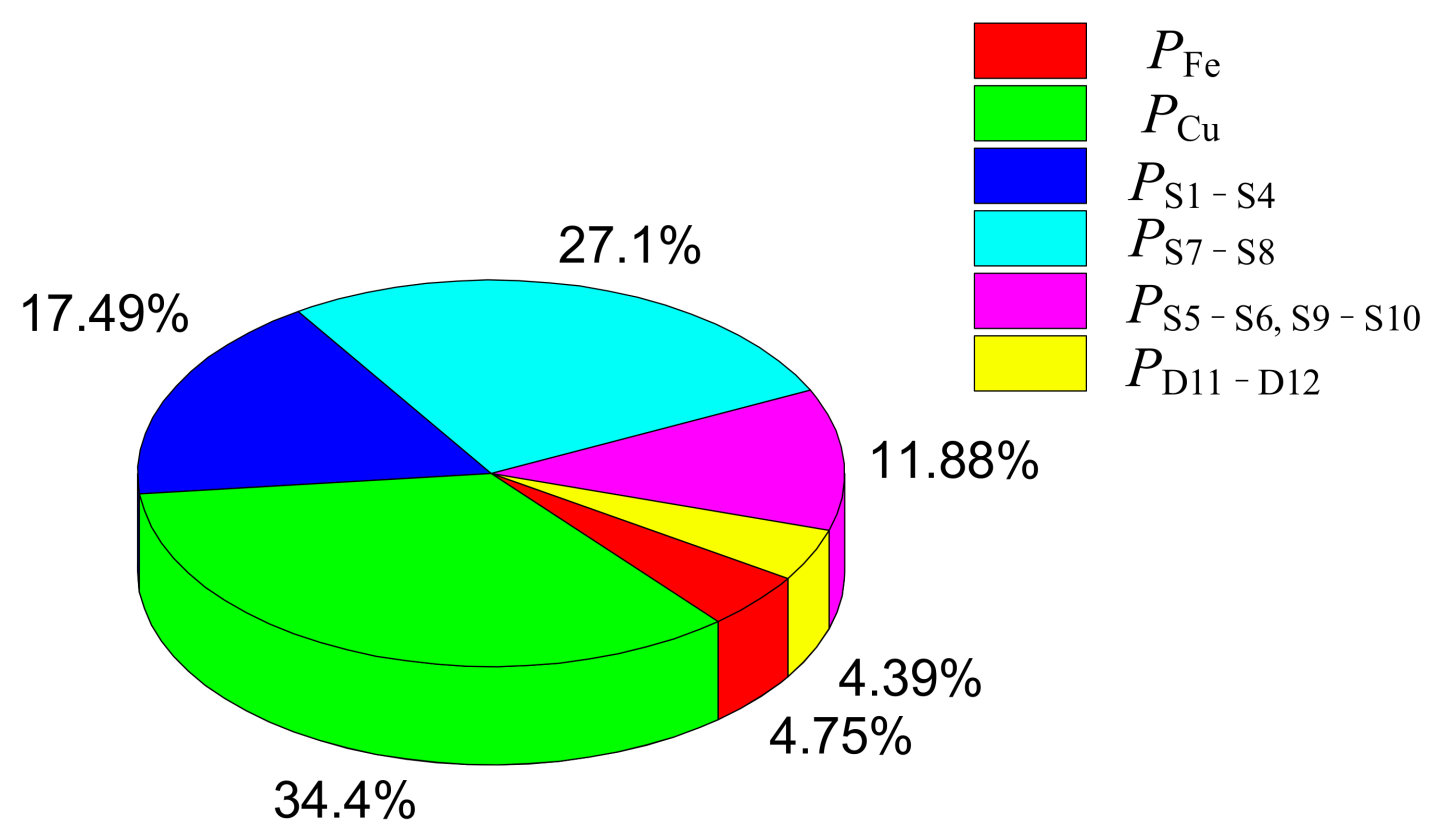
Loss breakdown of the proposed converter in CHG mode.

Figure 24 shows the loss breakdown of the converter in CHG mode. The transformer copper loss *P*_Cu_ is the dominant component, accounting for about 34.4% of the total loss. The losses of the Boost-arm switches S_7_–S_8_ and the LVS switches S_1_–S_4_ account for approximately 27.1% and 17.49%, respectively, while the losses of the HVS switches S_5_–S_6_ and S_9_–S_10_ account for about 11.88%.

In addition, the proposed converter is compared with several topologies reported in the literature, and the comparison results are listed in Table 5. The comparison indicates that the proposed converter provides a wide voltage-gain range within a relatively narrow frequency variation range, thereby enabling high-efficiency charging and discharging of batteries over a wide operating range. It should be noted that the reported efficiency values are not directly comparable due to differences in power ratings, voltage levels, switching devices, and experimental conditions among the converters.

## 6. Conclusions

A bidirectional DC–DC converter suitable for wide-range output applications is proposed in this paper. The converter provides two voltage gain modes for battery-discharging power transfer and three voltage gain modes for battery-charging power transfer. The main advantages of the proposed converter are summarized as follows:

1. A hybrid modulation strategy combining fixed-frequency PWM and PFM is adopted, with PWM as the primary operating mode, which simplifies the design of the magnetic components.
2. ZCS operation is experimentally verified at the representative operating points investigated in this study, contributing to reduced switching losses and improved converter efficiency.

The experimental results presented in this paper validate the feasibility of the proposed topology, its wide voltage-gain capability, ZCS characteristics under the tested operating conditions, and its efficiency performance. In addition, a simplified loss breakdown in CHG mode has been provided to identify the main loss contributions. Nevertheless, comprehensive device stress evaluation and detailed loss characterization across all operating modes and the entire operating range remain subjects for future investigation.

## Figures and Tables

**Table 1:** Comparison between calculated and measured voltage gains at representative operating points.

Mode	Calculated Gain	Measured Gain	Error
DMG	0.883	0.87	1.55%
DHG	1.197	1.19	0.58%
CLG	0.483	0.46	4.76%
CMG	0.885	0.84	5.10%
CHG	1.312	1.27	3.20%

**Table 2:** Design specifications of the proposed converter.

Operating Mode	Parameter	Value
Discharging operation	*U*_L_	40–120 V
	*U*_H_	200 V
	*I*_H_	3.75 A
Charging operation	*U*_H_	200 V
	*U*_L_	40–120 V
	*I*_L_	6.25 A

**Table 3:** Key parameters of the experimental prototype.

**Parameter**	**Symbols**	**Value**
DC bus voltage	*U*_H_	200 V
Battery voltage range	*U*_L_	40–120 V
Output power	*P*_o_	750 W
Switching frequency	*f*_s_	100 kHz
Resonant frequency	*f*_r_	100 kHz
Transformer turns ratio	*k*	2
Magnetizing inductance	*L_m_*	300 μH
Resonant inductance	*L*_r_	15.8 μH
Resonant capacitance	*C*_r_	160 nF
Switches	S_1_–S_4_	IRFP260NPBF
	S_5_–S_10_	NTHL110N65S3F
	D_11_–D_12_	CI30H60D3L2
Transformer core	T	PQ4040

**Table 4:** Representative measured efficiency values under different operating modes.

Operating Mode	Low-Power Point	Medium-Power Point	High-Power Point
CHG	462 W/91.19%	646 W/93.20%	728 W/90.69%
CMG	188 W/92.18%	596 W/95.23%	853 W/95.32%
CLG	150 W/93.51%	322 W/94.68%	407 W/93.50%
DMG	259 W/91.80%	576 W/94.51%	820 W/95.26%
DHG	250 W/87.24%	451 W/90.23%	743 W/89.99%

**Table 5:** Topology comparison.

Comparison		[12]	[15]	[17]	[19]	Proposed Converter
Topological structure	Number of power switches	4/4	8/6	6/6	10/0	10/2
	Number of transformers/resonant inductors/resonant capacitors	2/1/1	1/1/1	2/2/2	1/2/2	1/1/1
Modulation strategy		PWM	PFM	PFM	PFM	PWM + PFM
Power rating		1 kW	1 kW	250 W	500 W	750 W
Input/output voltage		400 V 160–320 V	390 V 60–450 V	50 V 50–300 V	400 V 250–450 V	200 V 50–120 V
Soft switching		ZVS	ZVS	ZVS	ZVS, ZCS	ZCS
Switching frequency range		Fixed frequency	Medium	Medium	Medium	Mainly fixed frequency
Bidirectional operation		No	No	No	Yes	Yes
Efficiency		94.2%	95.0%	94.8%	93.4%	95.4%

## Data Availability

The data presented in this study are available from the corresponding author upon reasonable request.

## Supplementary Materials

The following supporting information can be downloaded at https://www.mdpi.com/article/10.3390/s26175606/s1. Table S1: Summary of key component voltage and current stresses in CHG and DHG modes.
