# Supplementary material for: A Boost-Assisted Multi-Mode Bidirectional Resonant DC–DC Converter for Wide-Battery-Voltage-Range Applications
Source: Sensors (Basel). 2026 Sep 3;26(17):5606. doi: 10.3390/s26175606 (PMC13568170; doi:10.3390/s26175606)
Supplement: Supplementary file 1 [file sensors-26-05606-s001.zip › sensors-4447963-supplementary.pdf]

Table S1. Summary of Key Component Voltage and Current Stresses in CHG and DHG Modes.

| Operating mode | LVS bridge (S1–S4) | HVS resonant bridge (S5, S6, S9, S10) | Boost switches (S7, S8) | Diodes (D11, D12) | Lr | Cr | Transformer (T) |
|---|---|---|---|---|---|---|---|
| CHG（D=0.18, UL=120） | 120 V / 25 A | 200V / 15 A | 470V/15A | 530V/15A | 13 A / 210 V | 13 A /85 V | 440 V / 13 A |
| DHG（D=0.27, UL=84） | 85V / 56A | 200V / 28 A | 200 V /0 A | 200 V /0 A | 27 A / XX V | 28 A / 210 V | 200 V / 28 A |
